# Reelin regulates the migration and differentiation of extravillous trophoblastic cells

**DOI:** 10.1186/s40659-026-00690-1

**Published:** 2026-03-26

**Authors:** Nicole Sommer, Carlos Alarcon-Godoy, Héctor Pizarro, Aníbal Pacheco, Jorge A. Carvajal, Hugo Olguín, Jaime Gutiérrez, Andrea Leiva, María-Paz Marzolo

**Affiliations:** 1 Laboratorio de Tráfico Intracelular y Señalización, Facultad de Ciencias Biológicas, Pontificia Universidad Católica de Chile, 7810128 Santiago, Chile; 2 Departamento de Obstetricia, Facultad de Medicina, Pontificia Universidad Católica de Chile, 8331150 Santiago, Chile; 3 Laboratorio de Reparación Tisular y Células Troncales adultas, Facultad de Ciencias Biológicas, Pontificia Universidad Católica de Chile, 7810128 Santiago, Chile; 4 Facultad de Ciencias, Universidad San Sebastián, 7510602 Santiago, Chile

**Keywords:** ApoER2, EVT, Endothelial EVT, Hypoxia, Placentation, Preeclampsia, Trophoblast, Reelin, VLDLR

## Abstract

**Background:**

ApoER2/LRP8 is a receptor highly expressed in the placenta; however, its physiological role in this organ remains poorly understood. The VLDL receptor has been less studied in the placenta. Reelin, a relevant ligand for both receptors, is an extracellular glycoprotein that participates in neuronal polarization, migration and differentiation, hence having a central role in the central nervous system (CNS) development. Reelin triggers a complex signaling pathway that regulates cytoskeleton dynamics, cell migration, differentiation and gene expression. This paper aimed to determine whether the Reelin signaling pathway plays a role in cellular processes involved in placentation.

**Results:**

Reelin receptors ApoER2 and VLDLR were found in first-trimester extravillous trophoblast (EVT) cell lines. EVT cells Swan 71 responded to Reelin exposure by activating PI3K-Akt and increasing ApoER2 protein levels, but not VLDLR. Additionally, a dual role for Reelin via PI3K was established in these cells, as it enhanced trophoblastic migration at 2% O_2_ (to mimic the hypoxic physiologic conditions of trophoblastic migration) and promoted differentiation to an endothelial-like phenotype at 21% O_2_. Migration was also stimulated, independent of PI3K, when cells were exposed to normoxic (21% O_2_) or chemically induced hypoxic (CoCl_2_) conditions. We propose that, physiologically, during the first trimester, Reelin, together with its receptors, could stimulate trophoblastic migration and differentiation. Interestingly, under hypoxia, ApoER2 and VLDLR protein levels were increased, and Reelin modulated hypoxia-inducible factor HIF1-α levels. ApoER2, VLDLR and Reelin were detected in human term placentas from normal and pre-eclampsia with severe features (PE-SF) pregnancies. In the plasma of first-trimester pregnant women, Reelin levels were lower in patients with severe preeclampsia (PE) at a time before the onset of PE clinical symptoms.

**Conclusions:**

Reelin could be involved in placentation, playing roles in EVT migration and differentiation. The reduction in maternal Reelin levels detected early in pregnancy could be a potential biomarker for PE.

**Supplementary Information:**

The online version contains supplementary material available at 10.1186/s40659-026-00690-1.

## Backround

Reelin is a large extracellular matrix glycoprotein secreted during central nervous system (CNS) development [1–3], which regulates neuronal migration and positioning in the cortex, hippocampus and cerebellum. Reelin signals through receptors to the low-density lipoprotein receptor family, specifically LRP8/ApoER2 and VLDLR [3–5]. The canonical Reelin signaling cascade includes the recruitment and phosphorylation of the adaptor protein disabled-1 (Dab1) by Src-family kinases [6], followed by activation of phosphatidylinositol 3-kinase (PI3K), leading to activation of protein kinase B (Akt) [7], and by Rac1/Cdc42 branches [8]. Besides, p-Dab1 activates the branch of Crk and CrkL [9]. Independent of Dab1, Reelin activates extracellular signal-regulated kinases (Erk) via a non-canonical pathway [10]. Together, these and other signaling branches activated by Reelin promote several cellular responses, including cell migration, proliferation, and differentiation in the CNS and the periphery [3, 11, 12], with modifications in cytoskeletal dynamics, adhesion proteins, and gene expression [3].

The placenta is one of the tissues with the highest expression of ApoER2, with at least three receptor splice variants described [13, 14], one of which is detected only in placental mammals [15]. Until now, ApoER2 functions in the placenta have been associated only with its role in selenium transport [16–18] and in modulating the effects of antiphospholipid antibodies [19]. In contrast to ApoER2, information regarding VLDLR expression and function in the placenta is even scarcer, with its expression described several years ago by the group of J.F. Strauss [20]. The potential participation of Reelin signaling, along with its receptors, in normal or pathological placental development has not been thoroughly explored.

Proteomic analysis has revealed reduced levels of Reelin in maternal serum and in term-placental protein extracts from pregnancies suffering preeclampsia (PE) [21], one of the most lethal pregnancy-specific syndromes, compared to normal pregnancies [22]. PE is the leading cause of maternal and fetal mortality, and it is characterized by maternal hypertension, proteinuria, multiorgan damage, altered feto-maternal outcome, and the development of metabolic disorders later in life [22]. The most widely accepted hypothesis regarding the origins of PE is associated with reduced migration and invasion capacities of extravillous trophoblast cells (EVTs) [23]. In normal conditions, EVTs invade the maternal decidua from the implanting embryo to reach the uterine spiral arteries, where they acquire an endothelial-like phenotype, replacing the smooth muscle cells and endothelial cells of the uterine arteries, thereby increasing blood flow to the placenta and supporting fetal growth. Remarkably, EVT invasion through the maternal decidua occurs in a physiological low oxygen (O_2_) availability that extends from the seventh to the eleventh week of gestation, which is required for proper uterine artery remodeling [24].

Thus, defective invasion of EVTs during placentation has been proposed as the origin of PE and its maternal clinical features, such as endothelial dysfunction, hypertension, and end-organ dysfunction [22, 25]. Consequently, significant efforts have been directed towards understanding the regulation of EVTs invasion and its ability to remodel the uterine spiral arteries [26–28]. Since Reelin and its receptors have roles in cell migration, invasion, and differentiation, in addition to functioning as proangiogenic factors regulating of vessel wall integrity [3, 12, 29, 30], and these proteins have been found in the placenta, we aimed to investigate whether Reelin could regulate the migration of human EVTs as well as their ability to acquire an endothelial-like phenotype associated with placental development.

Here, we show, for the first time, that human EVT cell lines that express ApoER2 and VLDLR respond to Reelin exposure by activating the PI3K and Akt pathways. EVTs respond to Reelin treatment, enhancing migration and endothelial-like differentiation of EVTs cell lines through PI3K-dependent mechanisms. Given that PE is a pregnancy pathology characterized by altered trophoblastic migration, the surprising observation that Reelin is downregulated in first-semester plasma samples from women with preeclampsia with severe features (PE-SF) suggests that this glycoprotein may play a role in placentation and serve as a potential biomarker for PE development.

## Materials and methods

A detailed list of antibodies and oligonucleotides used in this study is in Supplementary Table S1 and S2.

### Human samples

Placental tissues were obtained from control (n = 9) or PE (n = 6) pregnancies at term (UC CHRISTUS Clinical Hospital, Santiago, Chile). For the control samples, gestational age ranged from 38 to 41 weeks. For PE samples, gestational age ranged from 37 to 40 weeks. From a different group of women, blood samples were obtained at the first trimester of pregnancy. These women were later diagnosed as having control (n = 5) or PE-SF (n = 5) pregnancies, according to the clinical criteria described later. The investigation was conducted in accordance with the ethical guidelines of the Declaration of Helsinki (Ethics approval from the Faculty of Medicine of the Pontificia Universidad Católica de Chile, ID 18081004). Informed consent was obtained from all the women, and clinical information was obtained from the medical staff. Preeclampsia (PE) was defined as new-onset hypertension (systolic blood pressure ≥ 140 mmHg or diastolic blood pressure ≥ 90 mmHg on two occasions at least 4 h apart after 20 weeks of gestation) accompanied by either proteinuria (≥ 300 mg in a 24-hour urine collection) or, in the absence of proteinuria, new-onset thrombocytopenia, renal insufficiency, impaired liver function, pulmonary edema, or new-onset cerebral or visual disturbances. Preeclampsia with severe features (PE-SF) was defined as systolic blood pressure ≥ 160 mmHg or diastolic blood pressure ≥ 110 mmHg, or the presence of any severe feature such as thrombocytopenia, acute renal failure, significant hepatic dysfunction, pulmonary edema, or persistent neurological symptoms [31]. The clinical characteristics of the women from whom the placentas were obtained are shown in Table S3, “Maternal and newborn clinical characteristics”.

### Cell culture

First-trimester EVT cells lines Swan 71 and HTR-8/SVneo were cultured in RPMI 1640 medium supplemented with 10% fetal bovine serum (FBS) and 1% penicillin (= 100 U/mL), 1% streptomycin (= 100 µg/mL) and 1% amphotericin B (= 0.25 µg/mL) and kept at standard conditions at 37 °C in a 5% CO_2_ incubator at 21% O_2_ (normoxia condition) or, in the case of Swan 71 also at 2% O_2_ (hypoxia). All the reagents were obtained from Thermo Fisher. hiPSCs-derived neurons (i3 Neurons) were cultured and differentiated as previously described in [32]. Briefly, iPSCs were cultured in Essential 8 medium (Gibco ^TM^ A1517001) supplemented with antibiotics (Gibco ^TM^ 15140163). When iPSCs reached 70–80% confluency, they were passaged and counted into Matrigel-coated (Corning 354277) dishes for neuronal pre-differentiation and then passaged, counted, and seeded onto poly-L-ornithine-coated (Sigma P3655) plates in the presence of Cortical Medium (BrainPhys Neuronal medium (STEMCELL Technologies 05790) with supplements). i3 Neurons were used after 30 days of differentiation.

### Recombinant Reelin

HEK 293 cells stably transfected to express the full-length mouse Reelin were used as described [33], and the recombinant protein was recollected from conditioned media. As a control, the mock-conditioned medium was prepared from HEK 293 cells, transfected with the empty vector, using the same protocol. To accomplish this task, confluent cells were cultured at 37 °C in a 5% CO_2_ incubator in high-glucose DMEM containing 10% FBS, penicillin, streptomycin, and 0.5 mg/mL G418 (Gibco, Thermo Fisher). After washing the cells twice with PBS, they were cultured in high-glucose DMEM without supplements for another 24 h. Cell-conditioned medium was collected for three consecutive days and centrifuged at 1850 x g for 5 min each time at room temperature. Finally, Amicon ultra-15 centrifugal filter units (filter membrane, 100 kDa) were used to concentrate the collected media by centrifugation at 880 x g for 10 min at 4 °C, until a final volume of 20 mL was reached. After collection, Reelin concentration was determined by running aliquots of medium and BSA samples (250, 500, 1000, 1500, 2000, and 2500 ng) on a 7% SDS-PAGE and staining with Coomassie blue. Protein bands were imaged using the Alliance Q9 system (UVITEC, Cambridge). Band intensities were quantified using ImageJ, and the BSA sample pixels were used to construct a standard curve. Reelin concentration was obtained by interpolating the band’s intensity with the BSA standard curve. Full-length Reelin (400 kDa) was quantified and taken as a reference to determine the final concentration of Reelin. For experiments, conditioned medium was diluted to 20 nM of Reelin.

### In vitro hypoxia induction

The 2% O_2_ approach was accomplished by incubating Swan 71 cells in an oxygen-regulated incubator and perfusing them with a gas mixture of 5% CO_2_ and 93% N_2_. In comparison, CoCl_2_ × 6H2O (Sigma-Aldrich C-2644) was prepared at a concentration of 200 µM in RPMI 1640 medium and supplemented to the cells during the experiments, while the cells were incubated at 21% O_2_. After completing the experiments, cells were rapidly frozen in liquid nitrogen and stored at -80 °C before lysis.

### Cell and placental lysis for protein determination

Swan 71, HTR-8/SVneo EVTs, and I3 neurons were washed with PBS and scraped from the plate with 80 µL of lysis buffer, then incubated on ice for 5 min. The RIPA lysis buffer used consisted of: 100 mM Tris-HCl pH 7.4 (Winkler 77861), 1% Triton X-100 (Sigma Aldrich 9002931), 0.5% Sodium deoxycholate (Sigma Aldrich 302954), 0.1% SDS (Calbiochem 151213), 1 mM EDTA (Winkler 6381926), 1 mM EGTA (Sigma Aldrich E-3889), 25 mM NaF (Winkler 7681494), 20 nM Na4P_2_O_7_ × 10 H_2_O (Sigma Aldrich S-9515), 5 mM Na_3_VO_4_ (Sigma Aldrich 13721396), 1.5 µM Aprotinin (Thermo Fisher 78432), 1 µM Antipain (Sigma Aldrich A6191), 1 µM Pepstatin A (Sigma Aldrich P5318), 50 mM PMSF (Thermo Fisher 329986), 50 µM Leupeptin (Santa Cruz SC295358), and 83 µM Benzamidine (Sigma Aldrich B-6506). Then, cells were centrifuged at 15,000 x g for 10 min at 4 °C. Finally, the supernatant was collected and quantified using the bicinchoninic acid (BCA) method (Thermo Fisher 23225).

Placental sections were homogenized to obtain protein extracts. Samples were lysed in solution 1 (10 mM EDTA, 50 mM Tris-HCl, pH 8.3, and an anti-protease cocktail) and mixed with an equal volume of solution 2 (4% SDS, 20% glycerol, 125 mM Tris/HCl, pH 6.8). The solution was then heated (50 °C, 10 min), sonicated (6 cycles, 10 s, 100 Watts, 4 °C), and spun down (15000 x g, 20 min) as described [34]. Finally, the supernatant was collected and quantified using the bicinchoninic acid (BCA) method (Thermo Fisher 23225) as described [34].

### Immunoblotting

Protein samples extracted from cells were prepared with loading buffer and denatured at 95 °C for 10 min before SDS-PAGE. The running conditions were set at 50 V for 20 min, followed by 100 V for 60–90 min at room temperature. Then they were transferred during 2 h at 0.3 A onto PVDF membranes (Thermo Fisher 88520), blocked with 5% BSA (5% w/v BSA in TBS Tween 20 0.1% v/v) or 5% non-fat milk (5% w/v milk in PBS Tween 20 0.1% v/v) and incubated with the corresponding primary-antibodies and subsequent HRP-conjugated secondary antibodies. Finally, images were obtained with the Alliance Q9 system (UVITEC, Cambridge) using the Westar Supernova kit (CYANAGEN XLS3,0100) and analyzed with ImageJ. Membrane stripping was accomplished by using a GnHCl solution [35] for 10 min.

### Immunofluorescence in cell lines

Coverslips containing Swan 71 cells were washed twice with PBS and fixed for 20 min at room temperature with 250 µL of 4% paraformaldehyde (PFA) and 4% Sucrose prepared in PBS. Afterwards, they were washed twice with PBS and permeabilized with 300 µL of 0.2% v/v Triton X-100 in PBS for 10 min at room temperature. Then, they were washed twice with 1X PBS and blocked with 30 µL of 5% BSA prepared in 1X PBS for 1 h in a wet chamber at room temperature, followed by inverted incubation with 20 µL of primary antibodies recognizing ApoER2 dissolved in 5% BSA overnight in a wet chamber at 4 °C. The coverslips were washed three times in 1X PBS and incubated for 1 h in a moist chamber at room temperature with 40 µL of secondary rabbit antibodies conjugated to Alexa Fluor 488 (Invitrogen), followed by 30 additional minutes in 1X Hoechst 33,342 (Invitrogen). Finally, cells were washed three times in 1X PBS and once in distilled water before mounting on slides with 9 µL of Fluoromount-G (495802, Invitrogen, Waltham, MA, USA). They were left to dry for 24 h before confocal microscopy (Nikon Eclipse Ti2, 60X objective). 12–15 z slices were obtained per image. All quantified image parameters were obtained using ImageJ version 1.8.0_322 software, and the nuclear-to-total ApoER2 ratio was calculated as the integrated density of nuclear ApoER2 divided by the integrated density of total cellular ApoER2 for each cell. The images were acquired using a Zeiss Airyscan confocal microscope (Zeiss, Germany) at the Microscopy Unit of the Pontificia Universidad Católica de Chile. Secondary antibodies were used as negative controls.

### Immunofluorescence in placental tissues

For immunolocalization in placentas (formalin-fixed, paraffin-embedded placenta), the samples were processed as described [34]. First, placental Sect. (5 μm) were incubated with anti-ApoER2, anti-CD31 (platelet endothelial cell adhesion molecule, PECAM-1 antibody), anti-cytokeratin 7 and anti-Reelin monoclonal E4 in blocking buffer. After rinsing with blocking buffer, the secondary antibodies Alexa Fluor 488 and 568 were incubated in blocking buffer, along with 0.1 mg/mL Hoechst 33,258 (Invitrogen) or DAPI. The images were visualized in the Pontificia Universidad Católica de Chile, UMA Microscopy Unit.

### Cell signaling assays

Swan 71 and HTR-8/SVneo cells were seeded in a 6-well plate and cultured for 24 h at 21% O_2_. Then, cells were washed with PBS, incubated at 21% O_2_ for 5 h with serum-free RPMI medium, and supplied with serum-free medium, mock, or 20 nM Reelin for 40 min. After cell lysis, proteins were analyzed using SDS-PAGE and immunoblotting.

### Half-life assays

Swan 71 cells were seeded into 6-well plates. After 24 h of incubation at 21% O_2_ in medium containing 10% FBS, cells were washed with PBS and exposed to 100 µg/mL cycloheximide (CHX) (Sigma Aldrich 66819) for 4 and 20 h at 21% O_2_. Lysed proteins were analyzed using the same SDS-PAGE and immunoblotting as described. The relative expression of ApoER2 and VLDLR was calculated as the ratio of each receptor, and matching β-actin signals, normalized to time 0 h, plotted, and analyzed with GraphPad after performing a logarithmic regression (y = − 0.183ln(x) + 1.1971 for ApoER2 and − 0.126ln(x) + 0.7046 for VLDLR). The receptors’ half-life was estimated as the time required for their levels to decline by 50% from their original levels.

### Biotinylation of cell surface proteins

Cells were washed with ice-cold PBS and biotinylated with 0.5 mg/mL of biotin for 15 min at 4 °C as described [32]. Then, biotin was quenched with 50 mM Tris, pH 7.5 and 100 mM NaCl twice for 10 min on ice. Cells were lysed with lysis buffer, centrifuged at 18,000 x g for 10 min, and incubated at 4 °C for 2 h with streptavidin beads (Pierce #20349) previously washed in lysis buffer. Beads were then washed once with PBS 1% Triton-X100, twice with PBS 1% Triton-X100 1 M NaCl and with a final PBS wash, centrifuging at 950 x g for 5 min at 4 °C. Finally, the beads were resuspended in loading buffer, and SDS-PAGE and immunoblotting were used to analyze the biotinylated proteins.

### Cell migration assays

Wound-healing assays were performed in Swan 71 cells. Briefly, on the reverse side of a 24-well plate, horizontal and vertical reference marks were generated in each well. Then, cells were seeded with 10% FBS medium and incubated for 48 h at 21% O_2_ to form a monolayer. Cells were washed with PBS and incubated in serum-free medium containing 1 µL/mL cytosine arabinoside (Ara-C) for 2 h. Subsequently, a six-pronged rake was dragged vertically across cells to form the wound, which was then washed with PBS to remove cellular debris. Finally, cells were incubated at 21% O_2_ for 24 h with mock or 20 nM Reelin with 1 µL/mL Ara-C. Using an epifluorescence microscope (Nikon Eclipse Ti-S) with a 20X objective and the NIS-Elements F program, bright-field images of the wound were taken in each well at 0 and 24 h, in the same position, using reference marks initially placed. For cell migration quantification at 24 h, the cell-free area was outlined and measured using the freehand selection tool in ImageJ software. This value was normalized to the initial wound area at zero time for each replicate, expressed as a percentage of the cell-free area. Total cell migration was defined as 100% minus the percentage of cell-free area. For the migration assay using chemical hypoxia induction, the same protocol was followed, with CoCl2 at 200 µmol/L administered alongside all conditions at the different time points. For 2% O_2_ hypoxia induction, cells were placed in a hypoxic incubator during all time intervals, but no images of the wound were taken at 0 h to prevent reoxygenation. The same protocol was replicated in parallel with 6-well plates to evaluate ApoER2 and HIF-1α levels by SDS-PAGE and immunoblotting. Finally, Swan 71 cells were treated with the PI3K inhibitor ZSTK474 at 0.1 µM and cell migration was evaluated under 21% O_2_ and hypoxia induced by 2% O_2_ or 200 µM of CoCl_2_, following the abovementioned indications.

### Formation of endothelial-like structures by trophoblast cells

This assay was conducted following protocols previously published by our group and others, with minor modifications [36–38]. In brief, 800,000 Swan 71 cells were seeded in 1 mL of RPMI-1640 supplemented with 1% FBS in 6-well culture plates containing 60 µL/cm^2^ of Cultrex basement membrane (R&D Systems) diluted to 70% in serum-free RPMI-1640 (Thermo Fisher). Specifically, conditioned media containing 20 nM of Reelin and mock media containing 1% FBS were added immediately after the cells were seeded. After 24 h, ten random phase-contrast microscopy images were captured using an Olympus CX41 microscope (Olympus, Tokyo, Japan) and digitized with a Moticam 2300 camera (Motic, Kowloon, Hong Kong). The cells’ ability to form two-dimensional endothelial-like structures was evaluated using the Angiogenesis Analyzer plugin in ImageJ (NIH), as previously described [37, 39]. The values for total length, number of segments, and branches of the formed endothelial-like structures were normalized to the Mock condition and presented as fold changes. For treatment with the PI3K inhibitor ZSTK474, cells were incubated with 0.1 µM inhibitor in 1.5 mL tubes for 30 min before seeding on the Cultrex basement membrane, as indicated.

### Real-time PCR

RNA extraction and qPCR assay were performed as described [32]. Total RNA was extracted from placental tissues and Swan 71 cells in RNAse-free conditions using TRIzol reagent (Invitrogen). 1 µg of RNA was reverse transcribed using RevertAid First Strand cDNA Synthesis Kit (Thermo Scientific #K1622). Real-time PCR was performed on a QuantStudio 3 Real-Time PCR System (Applied Biosystems, Thermo Fisher Scientific) with Hot FirePol Evagreen qPCR Mix and ROX (Solis Biodyne, #08-24-00001). The primers used for qPCR are described in Table S2. The qPCR conditions were denaturation at 95 °C for 15 s, annealing at 55 °C for 30 s and extension at 72 °C for 30 s. The expression levels of ApoER2 and VLDLR transcripts were normalized to GAPDH expression using the delta–delta Cq method (2^−ΔΔCq^).

In the case of the differentiation experiments of Swan 71 cells to eEVTs, the RNA was isolated using the E.Z.N.A.^®^ Total RNA Kit (Omega Bio-tek; Cat. No. R6834-01) according to the manufacturer’s instructions. RNA concentration and purity were assessed spectrophotometrically. Complementary DNA (cDNA) was synthesized from 300 ng of total RNA using the iScript™ cDNA Synthesis Kit (Bio-Rad), following the manufacturer’s protocol.

The relative expression levels of VE-cadherin and αV-integrin mRNAs were determined by quantitative real-time PCR (qPCR) using pre-designed TaqMan^®^ gene expression assays: Relative gene expression was calculated using the comparative Ct (ΔΔCt) method.

### Immunohistochemistry of placental tissues

For immunohistochemistry, placentas (formalin-fixed, paraffin-embedded placenta) were processed as follows: placental Sect.  (5 μm) were incubated with anti-reelin monoclonal antibody in blocking buffer overnight at 4 °C. After rinsing with blocking buffer, the secondary antibody anti-mouse HRP was incubated in blocking buffer 1 h at 37 °C. After rinsing with blocking buffer, diaminobenzidine was used for sample staining, and Mayer hematoxylin was employed for counterstaining. Images were captured using an Olympus CX41 microscope (Olympus, Tokyo, Japan) and digitized with a Moticam 2300 camera (Motic, Kowloon, Hong Kong).

### ELISA for Reelin determination

Blood samples were collected from pregnant women between 6 and 12 weeks of gestation, and they were grouped as preeclampsia with severe features (PE-SF) or controls according to the diagnosis obtained at 33 weeks of gestation. Aliquots of plasma were frozen (− 80 °C) after centrifugation of maternal blood. Plasma samples of 5 control women and 5 PE-SF women were analyzed. Reelin levels were determined in maternal plasma using ELISA (human Reelin ELISA kit, Cusabio) in two replicated assays.

### Statistical analysis

Statistical analyses were performed using GraphPad Prism software (version 8.0.2). Unpaired parametric two-tailed or one-tailed t-tests with Welch’s correction or one-way ANOVA were used for most experiments, and a confidence level of 95% was used for all tests. Most of the graphs were reported as mean ±SD (standard deviation) and some as mean ±SEM (mean standard error)), as indicated in the corresponding figure legend.

## Results

### ApoER2 and VLDLR, the Reelin receptors, are detected in trophoblastic cells and the placenta

**Fig. 1:**
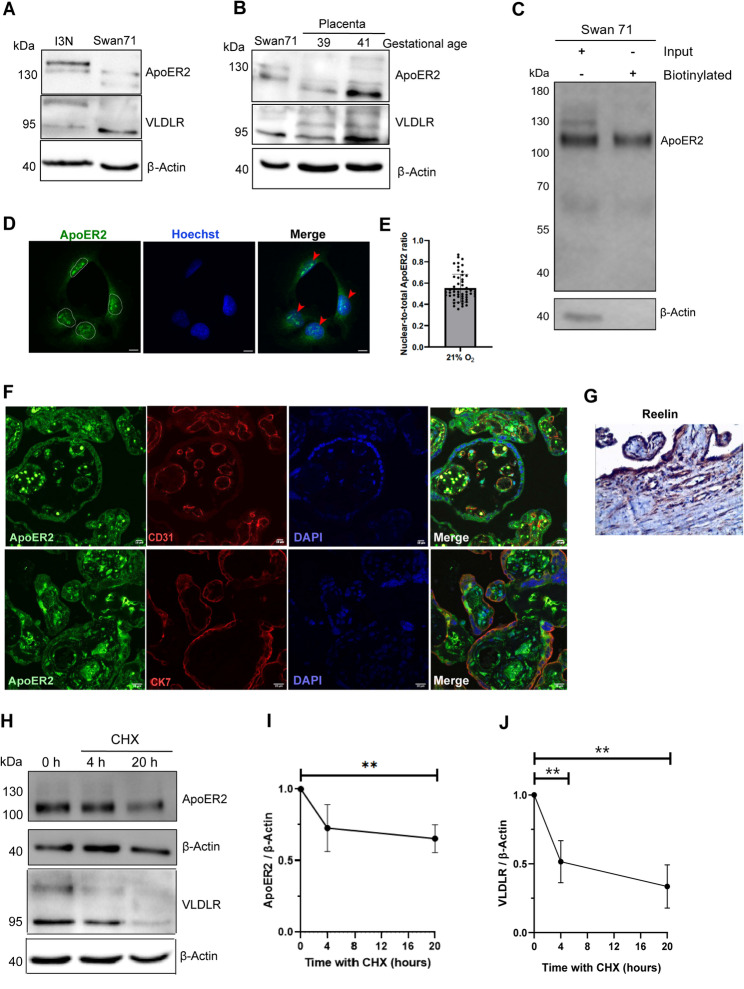
Expression of ApoER2, VLDLR and Reelin in normal human term-placentas and Swan 71 trophoblastic cell lines. **A** Immunoblot detection of ApoER2 and VLDLR present in protein extracts of Swan 71 cells grown in a medium supplemented with 10% FBS and human cortical neurons I3N as positive controls. 80 µg of protein were loaded. β-Actin was used as a loading control. **B** Samples of two different human term-placentas from normal pregnancies (gestational ages indicated as week + day) and Swan 71 cell extract were used to detect ApoER2 and VLDLR by immunoblot. 80 µg of protein were loaded. β-Actin was used as a loading control. **C** Total or cell surface (biotinylated) ApoER2 levels in Swan 71 cells treated overnight with serum-depleted RPMI medium, followed by cell surface biotinylation. 10% of the input (total receptor) and biotinylated fraction was loaded. **D** Confocal immunofluorescence of ApoER2 (green) present in Swan 71 cells cultured in 21% O_2_. Hoechst (blue) was used for nuclear staining and is shown by the white ROI outlines. Red arrows indicate ApoER2, possibly ICD, in the nucleus. Scale bar: 10 μm. **E** Quantification of the nuclear-to-total ApoER2 fluorescence levels ratio. The graph shows the mean ± SEM from three independent experiments, with 52 cells in total. **F** Human term-placentas, showing the presence of ApoER2 (green), the endothelial marker CD31(red), the trophoblast marker CK7 (red) and nucleus (DAPI). **G** Reelin expression in the human term placenta is shown by immunohistochemistry. **H** Representative western blot of ApoER2 and VLDLR protein levels for half-life determinations in Swan 71. Cells were incubated with complete RPMI medium containing 100 µg/mL CHX for 4 and 20 h, 80 µg of protein sample was loaded, and β-Actin was used as a loading control. **I**, **J** Quantification of ApoER2 and VLDLR half-life after different times of CHX treatment. The graphs represent mean ± SD of three independent experiments, and a two-tailed unpaired t-test was performed between the first and last time-points (** *p* ≤ 0.01)

The placenta is one of the tissues with the highest ApoER2 expression levels [13], yet its subcellular localization, detailed pattern and regulation mechanisms have not been fully described. On the other hand, the information about placental VLDLR is minimal. Our initial goal was to determine the presence of these proteins in first-trimester-derived EVTs cell lines and in normal human term placentas. First, we evaluated if both receptors were detected by immunoblot in one of the most used first-trimester-derived trophoblastic extravillous cell lines, Swan 71 cells (Fig. 1A) and in two samples of human term placenta of normal pregnancies (Fig. 1B). As a positive control for both receptors, we used a protein lysate of human neuronal cells i3N [32]. In Swan 71 ApoER2 displayed a distinct molecular band pattern compared to the neurons (Fig. 1A) and to placenta (Fig. 1B) probably reflecting the expression of different splice variants and proteolytic processing, both aspects that characterize ApoER2 in other systems like the CNS [40] and revealing potential differences in the receptor’s processing mechanisms or function between trophoblastic cells in culture and placental tissues. We detected VLDLR with a molecular weight more similar to that in neurons, Swan71, and the placenta (Fig. 1A, B).

To assess ApoER2 surface distribution in Swan 71 cells, we performed a biotinylation assay followed by immunoblotting (Fig. 1C). We detected a main biotinylated molecular weight band corresponding to the presence of ApoER2 on the surface of Swan 71 cells. The high-molecular-weight biotinylated bands (between 100 and 130 kDa) correspond to mature forms of the receptor and possibly to the full-length protein. ApoER2 was also detected on the surface of another widely used first-trimester cytotrophoblastic cell line, HTR-8/SVneo cells (Fig. S1A). Furthermore, ApoER2 was immunodetected in the cell nucleus of Swan 71 cells (Fig. 1D), where around 57% of the signal was localized (Fig. 1E) as determined by quantification of the nuclear-to-total ApoER2 ratio. This observation correlates with reports of the intracellular domain of ApoER2 (ICD), recognized by the antibody, which is induced and translocated to the nucleus in response to various stimuli, including Reelin [41–43].

ApoER2 immunoreactivity was detected in normal-term placentas, in both CD31- and CK7-positive cells, corresponding to endothelial and trophoblastic tissue compartments, respectively (Fig. 1F). Due to technical limitations with the antibody, we were unable to detect VLDLR using immunofluorescence. Regarding Reelin, one of the most relevant ApoER2 and VLDLR ligands, a positive signal for this protein was observed in normal human term placentas by immunohistochemistry (Fig. 1G), although its exact origin remains unknown.

To better understand the regulation of the receptors ApoER2 and VLDLR in Swan 71 cells, we assessed their degradation rates by exposing cells to 100 µg/mL cycloheximide (CHX) for 20 h (Fig. 1H). ApoER2 protein levels did not completely decrease during this period, suggesting an extended half-life of more than 20 h. Nonetheless, there was a significant drop in the receptor’s protein levels compared to its initial levels (Fig. 1I). In sharp contrast, VLDLR levels dropped almost to 50% after 4 h of CHX (Fig. 1J). These findings suggest that ApoER2 protein levels are more stable than those of VLDLR in Swan 71 trophoblastic cells.

Altogether, these results show that the ligand Reelin and the receptors ApoER2 and VLDLR are present in human placentas and, in the case of the receptors, in first-trimester-derived EVT cell lines. This evidence suggests that the components of the Reelin signaling pathway may be continuously present throughout pregnancy; however, potential variations in their placental expression across trimesters remain unclear.

### Characterization of Reelin signaling in extravillous trophoblast cells

**Fig. 2:**
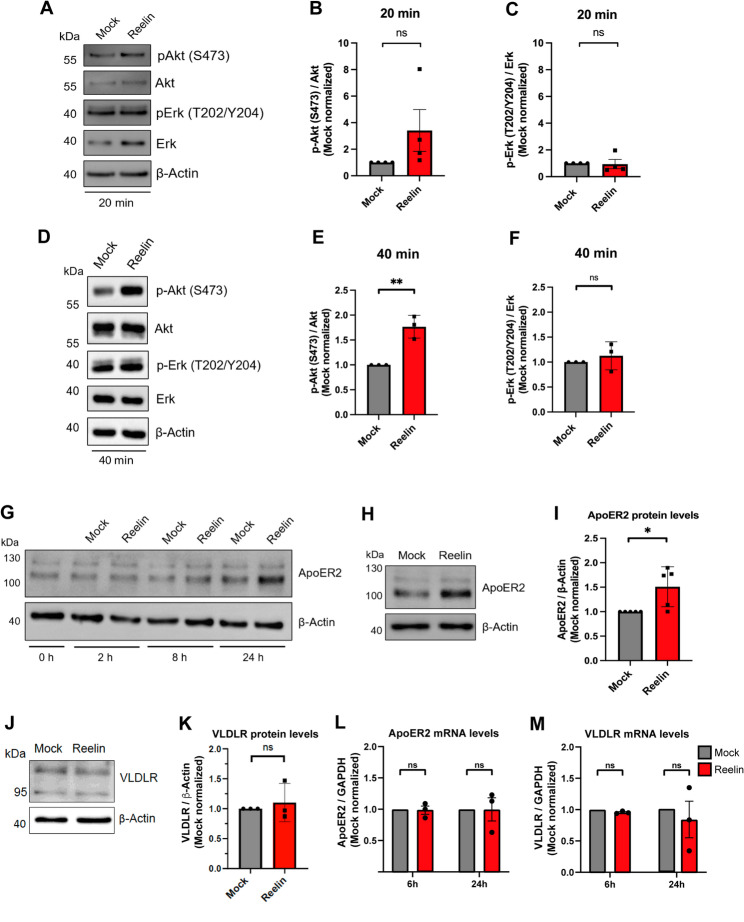
Swan 71 cells respond to Reelin by activating Akt. Swan 71 cells were treated with mock medium or 20 nM Reelin, cultured in 21% O_2_ for 20 and 40 min, lysed, and 40 µg of protein sample was loaded. β-Actin was used as a loading control. **A** Representative western blot and **B**, **C** quantification of total Akt, Erk, p-Akt (S473) and p-Erk (T202/Y204) levels detected after 20 min of stimulation. **D** Representative western blot and **E**, ** F** quantification of total Akt, Erk, p-Akt (S473) and p-Erk (T202/Y204) levels detected after 40 min of stimulation. Each graph corresponds to the mean ± SEM of four (A-C) and to mean ± SD three (D-F) independent experiments, and a two-tailed unpaired t-test was performed for all experiments (ns: *p* > 0.05 and ** *p* ≤ 0.01). **G** Immunoblot of ApoER2 in Swan 71 cells after 2, 8 and 24 h of exposure to mock or 20 nM Reelin in 1% FBS-supplemented medium. 80 µg of protein sample was loaded, and β-Actin was used as a loading control. **H** Representative western blot of ApoER2 levels after 24 h of exposure to mock or 20 nM Reelin, 80 µg of protein sample was loaded, and β-actin was used as a loading control. **I** Quantification of ApoER2 levels, normalized to mock values. The graph represents the mean ± SD of five independent experiments, and the data were analyzed by a two-tailed unpaired t-test (* *p* ≤ 0.05). **J** Representative western blot of VLDLR levels after 24 h of exposure to mock or 20 nM Reelin, 80 µg of protein sample was loaded, and β-actin was used as a loading control. **K**. Quantification of VLDLR levels, normalized to mock values. The graph represents the mean ± SD of three independent experiments, and the data were analyzed by a two-tailed unpaired t-test (ns: *p* > 0.05). Relative expression levels of **L** ApoER2 and **M** VLDLR mRNA obtained from Swan 71 cells exposed to mock or 20 nM Reelin for 6 and 24 h. The graphs correspond to mean ± SEM of three independent experiments, and relative expression levels were normalized to GAPDH expression using the 2^−ΔΔCq^ method. A two-tailed Mann-Whitney t-test was performed (ns: *p* > 0.05)

Next, we aimed to determine whether trophoblastic Swan 71 cells respond to Reelin treatments by activating the canonical and non-canonical signaling pathways previously characterized in neuronal cell types. The Reelin-induced activation was assessed by measuring the phosphorylation of Akt and Erk as described [32], using murine Reelin produced in conditioned medium from HEK293 cells transfected with a plasmid encoding Reelin; control (mock) medium was obtained from cells transfected with an empty vector. Murine Reelin use is supported by the high sequence homology between mouse and human Reelin (~ 94%) [44] and by evidence showing that murine Reelin effectively activates canonical Reelin signaling pathways in both human and mouse neurons [32]. Swan 71 cells were exposed to 20 nM of Reelin or mock medium for 20 (Fig. 2A) or 40 min (Fig. 2D). There was a trend, but not statistically significant, of increase in the phosphorylation levels of Akt (S473) after 20 min (Fig. 2B) that became significant after 40 min of Reelin stimulation (Fig. 2E). Erk phosphorylation levels were unresponsive to Reelin stimulation in any timeframe (Fig. 2C and F). These results indicate that Swan 71 cells respond to Reelin by differentially activating the Akt signaling branch of the pathway. Similar results were obtained with HTR-8/SVneo cells exposed to Reelin for 20 min (Fig. S1B–D) and 40 min (Fig. S1E–G).

Then, to better characterize the pathway in placenta-derived cells, we investigated whether Reelin receptor levels varied in response to different Reelin treatments. Swan 71 cells were exposed to 20 nM Reelin (or mock) for 2, 8 and 24 h (Fig. 2G), observing an increase in ApoER2 levels in cells treated with Reelin, compared to mock, after 24 h. We replicated the stimulation with Reelin for 24 h, resulting in a significant increase in ApoER2 levels compared.to mock (Fig. 2H, I), contrasting with no changes in VLDLR protein levels (Fig. 2J, K). In comparison, no changes in ApoER2 and VLDLR mRNA levels were observed after 6–24 h of stimulation (Fig. 2L, M). The specific effect of Reelin on ApoER2 protein expression, with no change in mRNA levels, suggests a previously unreported stimulatory feedback mechanism of Reelin on receptor levels in trophoblastic cells and reinforces differences in protein stability and regulation previously observed in half-life determinations.

### Reelin induces a PI3K-independent migration of Swan 71 cells

**Fig. 3:**
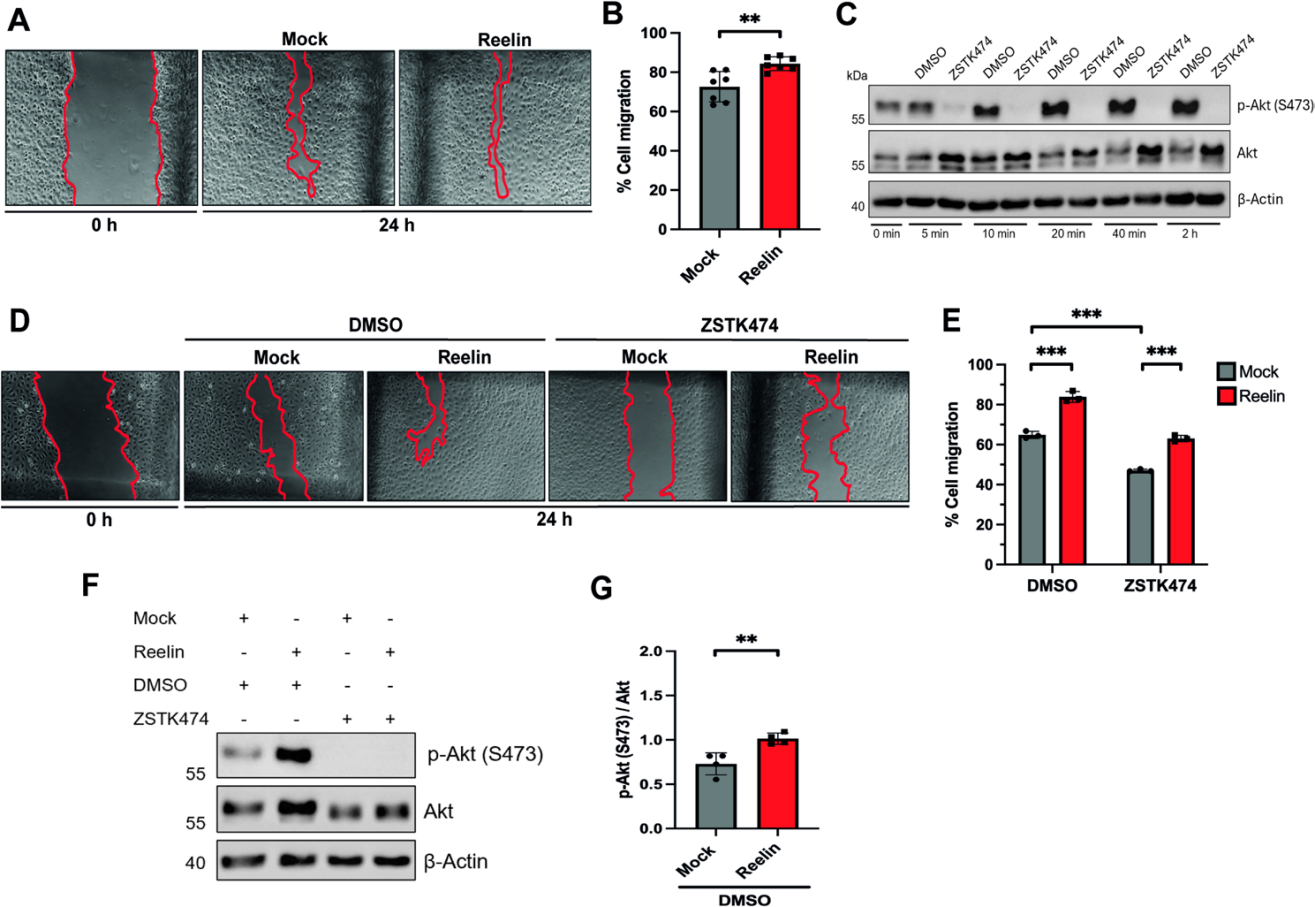
Reelin induces the migration of Swan 71 cells. **A** Representative images showing the wound-healing assay in Swan 71 cells before treatment (0 h) and 24 h after migration with mock or 20 nM Reelin medium. **B** Quantification of the percentage of cell migration in Swan 71 exposed to mock or Reelin. The graph shows the mean ±SD) migration percentage from seven independent experiments, and an unpaired two-tailed Welch’s t-test was performed (** *p* < 0.01). **C** Representative western blot of total Akt and p-Akt (S473) levels were detected in Swan 71 cells exposed to 5, 10, 20, 40 and 120 min with 0.1 µM ZSTK474 or DMSO in 1% FBS-supplemented medium, 80 µg of protein sample was loaded, and β-Actin was used as a loading control. **E** Representative images showing the wound-healing in Swan 71 cells before treatment (0 h) and after migration within 24 h of exposure to 0.1 µM of ZSTK474 or DMSO and mock or 20 nM Reelin medium. **E** Quantification of the percentage of cell migration in Swan 71 cells exposed to each condition. The graph corresponds to the mean ± SD of three independent experiments, and an unpaired one-tailed Welch’s t-test was performed (*** *p* ≤ 0.001). **F** Representative western blot showing total Akt and p-Akt (S473) levels detected in Swan 71 cells exposed to 24 h of 0.1 µM ZSTK474 or DMSO and mock or 20 nM Reelin, 80 µg of protein sample was loaded, and β-Actin was used as loading control. **G** Quantification of p-Akt (S473)/Akt levels is shown in DMSO-treated Swan 71 cells. The graph represents the mean ±SD) of four independent experiments, and an unpaired one-tailed Welch’s t-test was performed (** *p* < 0.01)

The migratory capacity of trophoblasts is a crucial function that these cells adopt in the early stages of human pregnancy to ensure proper placentation. In this regard, a relevant role of Reelin is the regulation of the migration of several cell types of the nervous system, including interneurons in the olfactory bulb [45], newborn neurons in the hippocampus, and cerebral cortex during development [5] and Schwann cells in the peripheral nervous system (PNS) [11]. Therefore, next, we evaluated whether Reelin could modulate the migration of Swan 71 cells. Briefly, using a wound closure assay, cells were allowed to migrate for 24 h after exposure to mock or 20 nM Reelin in a serum-free medium (Fig. 3A). To rule out the possibility that the wound could be closed by enhanced cell proliferation, we added 1 µg/mL Ara-C to all treatments, inhibiting the S phase of the cell cycle [46]. Reelin significantly increased the migration capacity of Swan 71 cells compared to the mock control (Fig. 3B), a novel role for Reelin in this cellular context.

In neurons and other cell types, such as Schwann cells, Reelin exerts its migratory effects through its receptors, triggering its canonical signaling, which involves the activation of Src, phosphorylation of Dab1, followed by the activation of PI3K [6, 7, 11]. Therefore, to determine whether PI3K plays a role in Reelin-induced migration of Swan 71 cells, we blocked PI3K with the Pan-Class I PI3K inhibitor ZSTK474 [47]. As a PI3K inhibition readout, we subsequently assess Akt inhibition. The phosphorylation of Akt, which reflects its activation, already decreased after 5 min of ZSTK474 exposure (Fig. 3C). Next, Reelin-induced trophoblastic migration was evaluated after 24 h of exposure to the PI3K inhibitor (Fig. 3D). In the control condition (mock media), Swan 71 migration was significantly decreased in PI3K-inhibited cells, compared to DMSO. In contrast, Reelin unexpectedly stimulated cell migration in ZSTK474 conditions (Fig. 3E). At the end of the 24 h of migration process, we measured the levels of Akt Ser-473; Reelin significantly increased p-Akt, as expected, when compared to the control (Fig. 3F, G), but this effect was abolished upon blockage of the Akt upstream activator PI3K (Fig. 3G). Overall, these results strongly suggest that Reelin-induced migration of Swan 71 cells occurs in a PI3K-Akt-independent manner.

### Reelin-induced responses exhibit differences under hypoxic conditions

**Fig. 4:**
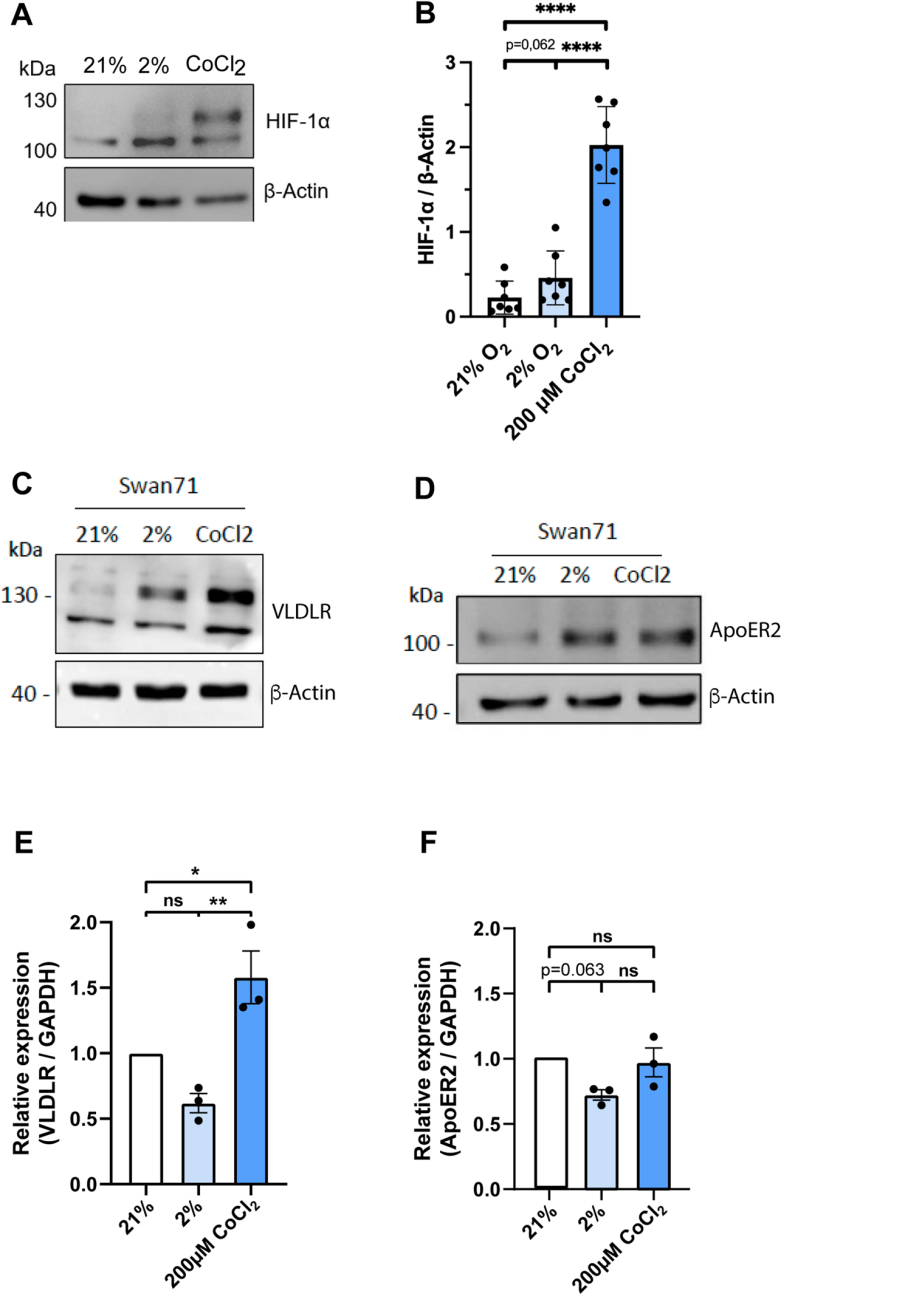
Effect of hypoxia on the expression of Reelin receptors ApoER2 and VLDLR. **A** Representative western blot of HIF-1α protein levels in Swan 71 cells cultured at 21% O_2_, 2% O_2_ or with 200 µM of CoCl_2_ during a period of 26 h with serum-depleted medium, 80 µg of protein sample was loaded, and β-Actin was used as a loading control. **B** Quantification of HIF-1α levels after exposure to 21% O_2_, 2% O_2_ or 200 µM of CoCl_2_. The graph corresponds to the mean ± SEM of seven independent experiments, and an unpaired two-tailed Welch’s t-test was performed (ns: *p* > 0.05 and **** *p* < 0.0001). **C** VLDLR and **D** ApoER2 immunoblot detection under the same conditions as shown in A. β-Actin was used as a loading control. **E**–**F** Relative mRNA expression levels of **E** VLDLR and **F** ApoER2 from Swan 71 cells cultured at 21% O_2_, 2% O_2_ or with 200 µM of CoCl_2_ for 6 h in serum-depleted medium. Gene expression levels were normalized to GAPDH and quantified using the comparative delta-delta Cq (2^(Δ−ΔCq)^) method. Data is presented as the mean ± SEM of three independent experiments, and one-way ANOVA was performed to calculate the statistical significance (ns: *p* > 0.05, *p < 0.05 and ** *p* < 0.01)

During the first trimester of pregnancy, the placental development involves trophoblast migration and invasion in a period of physiological hypoxia [24]. The induction of hypoxia largely depends on the activation of the hypoxia-inducible factor 1-alpha (HIF-1α) signaling pathway, as low oxygen tension stabilizes HIF-1α, leading to its nuclear translocation and regulation of genes encoding proteins necessary for oxygen homeostasis. At O2 levels below 21%, HIF-1α has a reported half-life of 5 min [48] due to rapid proteasomal degradation [49]. We aim to study Reelin responses in Swan 71 cells under hypoxic conditions, either chemically induced by 200 µM CoCl_2_ (an in vitro hypoxia-mimicking agent) or by exposure to 2% O_2_. To ensure proper hypoxia induction, HIF-1α protein levels were evaluated by immunoblot (Fig. 4A), revealing that HIF-1α was significantly accumulated, to a greater extent with 200 µM CoCl_2_ than with 2% O_2,_ compared to 21% O_2_ (Fig. 4B). Interestingly VLDLR (Figs. 4C and S1H in HTR-8/SVneo cells) and ApoER2 (Fig. 4D) protein levels were evidently increased under the same hypoxic conditions, for 26 h, however only in the case of VLDLR (Fig. 4E) but not of ApoER2 (Fig. 4F) we found a significant increase in the mRNA levels after 6 h of CoCl_2_ treatment.

**Fig. 5:**
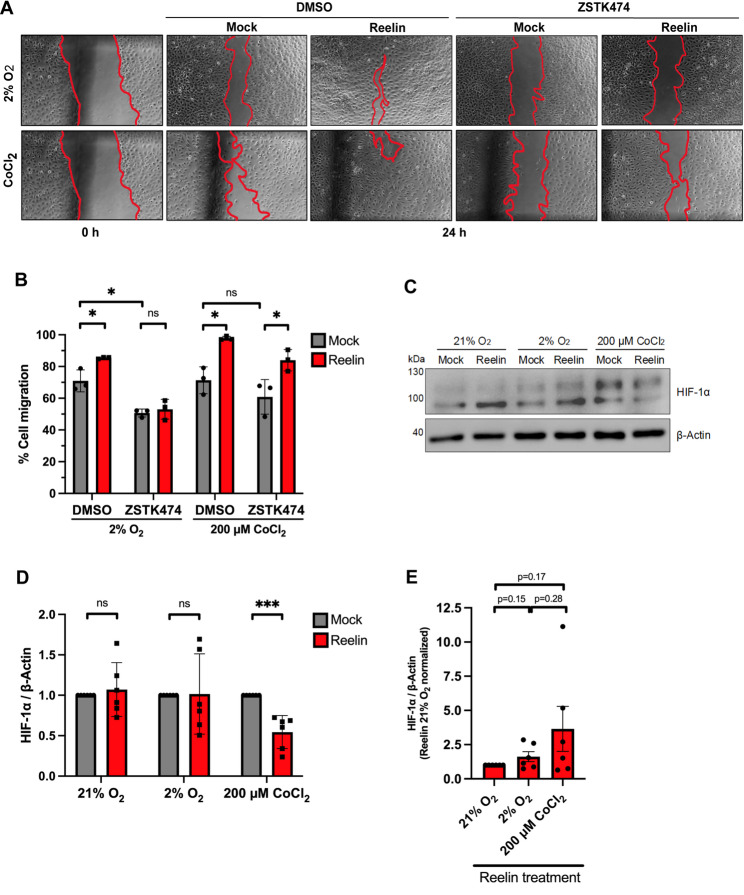
Migration of Swan 71 cells in response to Reelin under hypoxia conditions. **A** Representative images showing wound-healing assay in Swan 71 cells before treatment (0 h) and after migration within 24 h of exposure to 0.1 µM of ZSTK474 or DMSO and mock or 20 nM Reelin medium in 2% O_2_ or 200 µM of CoCl_2_. **B** Quantification of the cell migration in Swan 71 cells of the experiment is shown in A. Graph corresponds to mean ± SD of three independent experiments, and an unpaired two-tailed Welch’s t-test was performed (ns: *p* > 0.05 and * *p* ≤ 0.05). **C** Representative western blot of HIF-1α is shown in cells cultured at 21% O_2_, 2% O_2_ or 200 µM of CoCl_2_ for 26 h with serum-depleted medium containing mock or 20 nM Reelin medium. 80 µg of protein sample was loaded, and β-Actin was used as a loading control. **D** Quantification of HIF-1α levels after exposure to 21% O_2_, 2% O_2_ or 200 µM of CoCl_2_ in the presence of Reelin (or mock) and **E** Quantification of HIF-1α levels after exposure to 21% O2, 2% O2 or 200 µM of CoCl2 in the presence of Reelin, normalizing the data to the 21%O2 condition. The graphs correspond to the mean ± SD of six independent experiments, and an unpaired two-tailed Welch’s t-test was performed (ns: *p* > 0.05 and *** *p* < 0.001)

Therefore, Reelin-induced wound closure assays and the determination of the requirement for PI3K in trophoblast migration were additionally performed in Swan 71 cells under hypoxic conditions. Reelin significantly stimulated the process in both hypoxic contexts (Fig. 5A, B). Nonetheless, upon PI3K inhibition with ZSTK474, Reelin only stimulated Swan 71 cell migration in the presence of CoCl_2_, an effect abolished in cells incubated at 2% O_2_ (Fig. 5A, B). As in the normoxic condition, Akt Ser-473 phosphorylation levels were undetectable upon PI3K inhibition in both hypoxic conditions, 200 µM CoCl_2_ (Fig. S2A) and 2% O_2_ (Fig. S2C). Surprisingly, and in contrast to the normoxic context (Fig. 3F, G), in the control (DMSO) condition, p-Akt in Ser-473 mainly remained unchanged between Reelin and mock exposures in 200 µM of CoCl_2_ (Fig. S2B) and 2% O_2_ (Fig. S2D) context, indicating that Reelin cannot fully activate Akt under hypoxic conditions. Interestingly, HIF-1α protein levels were decreased in cells treated with CoCl_2_ in the presence of Reelin for 26 h (Fig. 5C, D) to levels similar to those in 2% O_2_ (Fig. 5E), indicating that this pathway regulates hypoxia.

These results indicate that Reelin-induced migration of EVT cells depends on PI3K under low O_2_ tension, which corresponds to the hypoxia induction method that best simulates in vivo physiological conditions during the early stages of placenta development, when migration occurs. In conclusion, these results suggest that Reelin significantly enhances the migration of EVTs in both low- and control-oxygen conditions, although through different pathways: PI3K-dependent or PI3K-independent, respectively.

### Reelin stimulates endothelial trophoblastic differentiation in a PI3K-dependent manner

**Fig. 6:**
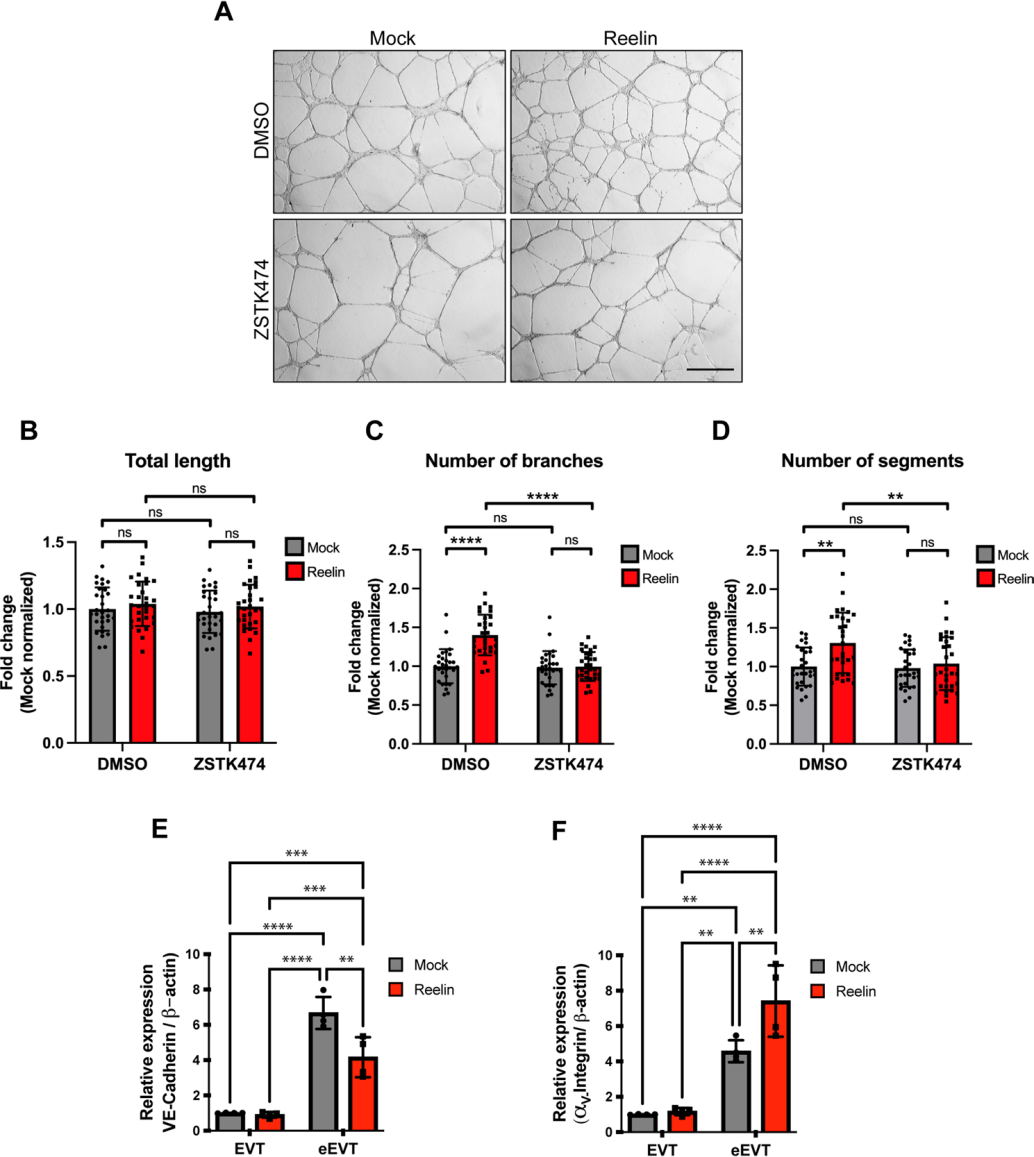
Reelin promotes the branching of tube-like structures formed by Swan71 cells in a PI3K-dependent mechanism and modulates endothelial marker expression. **A** Representative images of Swan71 cells after 24 h of being seeded over pre-polymerized Cultrex basement membrane and cultured under normoxic standard conditions in the presence or absence of 20 nM Reelin and/or 0.1 µM of the PI3K inhibitor (ZSTK474) in a 1% FBS-supplemented medium. Scale Bar 200 μm **B** Quantification of the total tube length (total length), **C** number of branches and **E** number of segments of the tube-like structures shown in A were analyzed by the angiogenesis analyzer plugin of NIH ImageJ software. Ten random pictures were taken from each assay from three independent experiments. Statistical differences were analyzed by a one-way ANOVA (ns: *p* > 0.05, ** *p* < 0.01 and **** *p* < 0.0001), and all values correspond to mean ± SD. **E**, **F** Relative mRNA expression of VE-cadherin and αv-integrin quantified by qPCR and normalized to β-actin. Matrigel culture increased the expression of both markers. A two-way ANOVA analyzed statistical differences with Tukey’s multiple comparisons test (* *p* < 0.05, ** *p* < 0.01, *** *p* < 0.001 and **** *p* < 0.0001), and all values correspond to mean ± SD from four independent experiments

It has been reported that hypoxia inhibits trophoblastic differentiation from extravillous trophoblasts (EVT) to the endothelial EVT (eEVT) phenotype [50], whereas normoxia enhances this process. Therefore, we then conducted a differentiation assay using Swan 71 cells. These cells were exposed to either Reelin or a mock treatment on a Cultrex membrane exclusively under normoxic conditions for 24 h (Fig. 6A). To investigate whether the PI3K pathway contributes to developing these endothelial-like structures, we co-incubated the cells with either the PI3K inhibitor ZSTK474 or a DMSO control, as we did in the migration assay. We observed that the total tube length formation in Swan 71 cells remained unchanged across all conditions (Fig. 6B), indicating that this process was independent of Reelin and PI3K. However, Reelin treatment significantly increased the number of branches (Fig. 6C) and segments (Fig. 6D) formed between cells compared to the control (mock), effects that were abolished by PI3K inhibition. These findings indicate that Reelin enhances the branching of endothelial-like Swan 71 cells via a PI3K-dependent mechanism, suggesting a role in endothelial differentiation into eEVTs.

To corroborate and further characterize the impact of Reelin on the endothelial transition of EVT cells, we analyzed VE-cadherin and αv-integrin expression by qPCR in Swan 71 cells cultured under EVT conditions or after endothelial-like differentiation (eEVT) on Matrigel. As expected, culture on Matrigel significantly increased the expression of both VE-cadherin and αv-integrin compared to EVT conditions, regardless of treatment (Fig. 6E, F). However, Reelin differentially modulated the expression of these markers in eEVT cells. While VE-cadherin expression was significantly induced in eEVT cells, its expression levels were markedly lower in the presence of Reelin compared to mock-treated cells (controls) (Fig. 6E). In contrast, αv-integrin expression was further increased in Reelin-treated eEVT cells relative to mock conditions (Fig. 6F). No significant differences in VE-cadherin or αv-integrin expression were detected between mock- and Reelin-treated cells under EVT conditions. These results indicate that Reelin selectively alters the expression balance of endothelial-associated markers during EVT endothelial differentiation, reducing VE-cadherin induction while enhancing αv-integrin expression in eEVT cells.

### Reelin and its receptors in preeclampsia

**Fig. 7:**
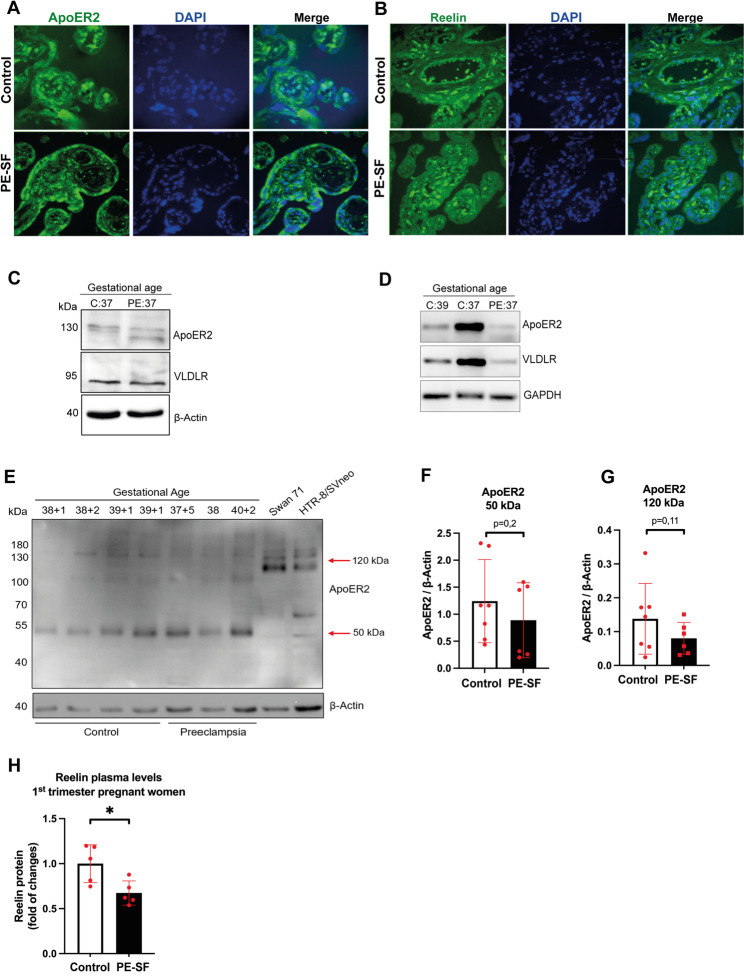
ApoER2, VLDLR and Reelin are expressed in PE-SF placentas. **A** Immunofluorescence showing ApoER2 in green and DAPI (blue) for nuclear staining on histological sections from human PE-SF and normal term placentas (control). **B** Immunofluorescence showing Reelin in green and nuclear staining (DAPI) in blue, on histological sections from human PE-SF and normal term placentas (control). **C** Representative immunoblot for ApoER2 and VLDLR from one control and one PE-SF term-placenta. β-Actin was used as a loading control. **D** Agarose gel electrophoresis of PCR products for ApoER2, VLDLR, and GAPDH mRNAs from two controls and one PE-SF human placentas. **E** Representative western blot of ApoER2 from term-placentas of women with different gestational ages (week + day) of control (38 + 1, 38 + 2, 39 + 1 and 39 + 1) and preeclamptic (37 + 5, 38 and 40 + 2) pregnancies or Swan 71 cells and HTR-8/SVneo cultured in 10% FBS-supplemented medium. 80 µg of protein was loaded, and β-Actin was used as a loading control. **F** Quantification of ApoER2 molecular weight bands of 120 kDa and **G** 50 kDa are shown. All graphs represent the mean ± SD of 7 samples for preeclampsia and 6 for control placentas, and an unpaired one-tailed Welch’s t-test was performed (ns: *p* > 0.05). **H** Plasma Reelin levels were determined by ELISA in 6- and 12-week maternal plasma samples collected from 5 first-trimester PE-SF patients and 5 control patients. Samples were grouped as PE-SF or control according to the diagnosis obtained at 33 weeks. The graph represents mean ± SD, and a one-tailed Mann-Whitney t-test was performed (* *p* ≤ 0.05)

PE is a pregnancy pathology that is caused by a chronic state of hypoxia that leads to poor placentation [51]. Our in vitro experiments using the first-trimester-derived EVTs Swan 71 suggested changes in Reelin receptor expression under hypoxic conditions. Since we detected ApoER2 and VLDLR in normal (control) human term-placentas (Fig. 1B), we confirmed through immunofluorescence that ApoER2 (Fig. 7A) and Reelin (Fig. 7B) were also expressed in pathological term-PE-SF placentas of similar gestational age. Additionally, VLDLR and ApoER2 protein and mRNA were detected in placental extracts from controls and one pre-eclamptic term placenta from similar gestational ages (Fig. 7C, D). Besides, in a different set of placenta samples, we were able to immunodetect ApoER2 it in its full-length size and other fragments (Fig. 7E), finding that despite a trend towards lower ApoER2 protein levels in PE term-placentas, the reduction was not statistically significant compared to control pregnancies for either 50 kDa (Fig. 7F) or 120 kDa (Fig. 7G) receptor molecular weight bands. Our results confirm that Reelin and its receptors are expressed in human term placentas. Still, our experiments do not allow us to conclude whether there are differences in the expression of these proteins in placentas from normal and PE pregnancies.

Finally, to determine whether Reelin protein levels differ between normal and PE pregnancies before the development of PE clinical features, circulating Reelin was measured by ELISA in first-trimester plasma from a second cohort of pregnant women. A group of them were later diagnosed with PE-SF in their second trimester. Surprisingly, Reelin levels were significantly lower in the plasma of PE-SF patients than in control pregnancies (Fig. 7H). Reduced Reelin levels in the plasma of mothers who will develop PE may imply a role for the Reelin signaling pathway in the development of PE, due to defects in EVT migration and proper differentiation into eEVTs.

## Discussion

Since the discovery of ApoER2/LRP8, the Reelin receptor, its high expression in the placenta has been reported in both human and mouse [13, 14]. In contrast, the information related to VLDLR function in the placenta is relatively scarce; it has only been suggested as a lipoprotein/lipid transporter [20]. Moreover, some evidence for the role of ApoER2 in the placenta has been proposed, suggesting that it acts as a receptor for Selenoprotein P [16, 17, 52], consistent with the neuroprotective role of ApoER2 against neurodegenerative disorders [53], which are characterized by selenium imbalances [54]. Placental ApoER2 has also been given a pathological denomination linked to the antiphospholipid (aPL) syndrome, an autoimmune condition associated with pregnancy loss [55]. It was reported that upon trophoblastic exposure to antiphospholipid antibodies, ApoER2’s interaction with the membrane glycoprotein β2-GPI was required for the pathological impairment of trophoblastic proliferation, invasion and migration capabilities [19]. However, a question arises: What other cellular physiologic and non-pathologic processes, besides selenium uptake, could ApoER2’s expression influence in normal placentas during pregnancy? Despite the expression of ApoER2 and VLDLR, no role for Reelin has yet been determined for placenta development and function. Therefore, we aimed to characterize the Reelin signaling pathway, including its receptors, in the human placenta and in cell lines derived from early EVTs.

### EVTs respond to Reelin by increasing their migration and differentiation, which are relevant processes for placentation

Our work’s relevant and novel findings include the role of Reelin and its induced responses in EVTs. First, we showed that after Reelin exposure, Swan 71 and HTR-8/SVneo cells activate Akt (but not Erk). Second, as will be discussed in detail, Reelin induces the migration and differentiation of EVTs. In neurons, Reelin activates its canonical signaling pathway depending on Dab1 phosphorylation [56, 57]. Nevertheless, it is important to note that Dab1 is not expressed in human trophoblastic cell lines or in human and mouse placenta extracts [58]. The involvement of an alternative adaptor protein in mediating Reelin’s responses warrants further investigation. In this context, the endocytic/signaling adaptor Dab2 emerges as a potential candidate, given that its interaction with ApoER2 has already been described [59]. As ApoER2, Dab2 is associated with trophoblastic dysfunction in the antiphospholipid syndrome upon aPL exposure [58] and regulates leucocyte adhesion to endothelial cells in a PI3K-Akt-dependent manner [60]. Moreover, Dab2 is required for HTR-8/SVneo migration [61]. In neurons and Schwann cells [7, 11], Reelin-induced migration depends on PI3K, Cdc42, and Rac1 and modifies actin cytoskeleton dynamics [8]. Interestingly, in our wound-healing migration assays, Reelin induced a significant EVT migration; however, this process was independent of PI3K-Akt under control culture conditions (21% O_2_). Still, several possibilities could explain this Reelin-induced migration, including the involvement of Rac1, as has been observed in other cell types. On the one hand, ApoER2 interacts with the polarity complex protein Par3 and, in turn, recruits Tiam1, a Rac1 GEF, thereby mediating Reelin-induced Schwann cell migration [11]. Also, Rac1 can be activated via the Dock180-ELMO complex, as shown in several systems, including migrating endothelial cells [62]. These and other possible mechanisms will be evaluated in the near future.

As discussed, EVTs are placental cell types that have been described to migrate during the first trimester of pregnancy, mainly in a physiological period of hypoxia that extends from the seventh to the eleventh week of gestation and involves a temporary decrease in oxygen (O_2_) availability from 8% to 2% [24]. Then, we were interested in disclosing the role of Reelin in conditions mimicking physiologic hypoxia that occurs during the first weeks of human pregnancy. Akt activation and migration were measured in 2% O_2_ and under chemically induced hypoxia with CoCl_2_ (a condition in which the cells are cultured in the presence of normal levels of O_2_). To validate hypoxia, we detected HIF-1α, which was more evident under chemical hypoxia induced by 200 µM of CoCl_2_, as this agent stabilizes HIF-1α by directly inhibiting hydrolytic enzymes that would otherwise cause its proteasomal degradation in normoxic conditions [49, 63]. Interestingly, only under low oxygen (2%) [64], the Reelin-induced migration capacity of trophoblastic Swan 71 cells was PI3K-dependent. Therefore, irrespective of O_2_ or HIF-1α levels, Reelin exposure stimulated migration of first-trimester EVTs. The dual mode of Reelin-induced responses was surprising; however, the literature shows that a decrease in oxygen tension up- and down-regulates a series of genes in trophoblasts [23], including the oxygen sensor HIF-1α, making them more prone to migration/invasion. These changes could underlie the differential Reelin dependency on PI3K to exert its effects. Besides, Reelin itself could be related to hypoxia, as suggested in different systems. Our findings showed that trophoblast cells exposed to chronic CoCl_2_, known to be toxic under some circumstances via elevated levels of HIF-1α [65], if they were co-incubated with Reelin, exhibited a significant reduction in HIF-1α to levels close to 2% O_2_, revealing a novel relationship between hypoxia and Reelin in the human placenta. Few pieces of evidence demonstrate associations between Reelin and hypoxia. For instance, in normoxia, treating human melanoma cell lines with recombinant Reelin induced HIF-1α through the PI3K/Akt signaling pathway [66]. In contrast, during cortical development, HIF-1α knockout mice exhibited cortical layer disorganization [67], which correlated with the phenotype observed in *reeler* mice [68]. Moreover, prenatal exposure to 9% O_2_ reduced Reelin levels in the brains of heterozygous *reeler* mice [69]. Therefore, both proteins could be regulated by a feedback mechanism in the placenta, which would require further investigation.

EVTs’ migration and invasion enable the anchoring of the fetus to the mother and the remodeling of the uterine spiral arteries, ensuring proper gas and nutrient exchange throughout the rest of the pregnancy [23, 70, 71], promoting a normoxic environment that is complete by week 22 [23]. During this process, some EVTs differentiate into an endothelial phenotype (eEVT). This evidence prompts us to question whether Reelin could, in addition to influencing trophoblastic migration, regulate EVT differentiation to eEVT. As an approach to study differentiation, we used a protocol indicative of its transformation to eEVT in 21% O_2_ [36–38]. Reelin treatment induced a significant increase in the branching process in a PI3K-dependent manner, suggesting a role in endothelial differentiation. Although Reelin did not increase the overall capacity of Swan-71 cells to form tube-like structures on Matrigel, our qPCR data provide mechanistic insight into how Reelin modulates the quality of the EVT-to-endothelial transition. Culture on Matrigel induced VE-cadherin and αv-integrin expression independently of treatment, consistent with activation of an endothelial-like differentiation program. However, Reelin reshaped this response by attenuating VE-cadherin induction while further enhancing αv-integrin expression. VE-cadherin is a central regulator of endothelial cell–cell junction stabilization and vessel maturation, and its sustained upregulation is associated with reduced motility and termination of angiogenic sprouting [72]. In contrast, αv-integrins are markers of angiogenic vascular tissue [73], facilitating the adhesion and migration of endovascular trophoblasts within uterine arteries [74]. In endothelial cells, angiogenic activation is characterized by a relative reduction or redistribution of VE-cadherin-mediated adhesion, accompanied by increased αv-integrin signaling. This molecular balance favors sprouting and branching rather than vessel stabilization [75]. This signature closely mirrors our functional observations, in which Reelin increased branching complexity without affecting total tube length, a phenotype widely interpreted as active angiogenic remodeling rather than endothelial maturation. In the placental context, αv-integrin upregulation defines invasive and endovascular EVT during spiral artery remodeling, whereas premature or excessive VE-cadherin stabilization is incompatible with the plastic, remodeling-competent phenotype required for this process [76, 77]. Importantly, defective EVT plasticity, impaired endovascular remodeling and reduced angiogenic branching are hallmarks of preeclampsia, particularly in early-onset and severe forms of the disease. Thus, our data support a model in which Reelin promotes a dynamic, angiogenic-like eEVT state that is physiologically required for spiral artery transformation, and whose disruption may contribute to the shallow placentation and vascular dysfunction characteristic of preeclampsia.

In this regard, it is relevant to note that Reelin, via ApoER2, induces the expression of adhesion molecules on endothelial cells, thereby increasing leukocyte interaction [60, 78]. This pathway depends on Dab2 (not Dab1, which is like what would eventually happen in the placenta, lacking Dab1), Akt, and NF-kappaB. Another study assesses lymphatic vasculature formation and the interaction between smooth muscle cells and lymphatic endothelium. In this case, the endothelium produces Reelin, which increases in response to interaction with smooth muscle cells and functions as a chemoattractant [79]. A similar theory could be proposed regarding the interaction between trophoblasts and the endothelium in the placenta during spiral artery remodeling. In this regard, it has been shown that the expression of Dab2 in EVTs is critical for orchestrating the phenotypic transition and motility of decidual vascular smooth cells induced by chemokine CXCL8 (IL8) in a PI3K/Akt pathway [61], again underscoring the role of this adaptor and PI3K in uterine spiral arteries remodeling.

Another finding was that exposure of cells to hypoxia decreased Akt total protein levels and activation, as observed in other cell types, such as HepG2, over long periods [80]. Therefore, prolonged hypoxia completely abolished its mTOR-dependent Akt phosphorylation and complete activation. Coincidentally, a report suggests that the Akt-mTOR signaling pathway is also downregulated in conditions of HIF-1α accumulation, as observed in HTR-8/SVneo cells treated with CoCl_2_ [81]. Nonetheless, multiple reports suggest contradictory effects of hypoxia on Akt levels, induced by mimicking agents or hypoxia alone, which are probably due to cell-type differences and differences in the timing of exposure to hypoxic conditions [80]. In our system, Reelin induced EVT migration under 2% O_2_ or CoCl_2_-induced hypoxia, in a PI3K-dependent or independent manner, respectively, but in both cases, Akt was not fully activated.

Conversely, the dual role of Reelin, promoting hypoxic trophoblastic migration and enhancing trophoblastic differentiation in normoxia, aligns with evidence indicating that prolonged hypoxia hinders trophoblast differentiation into an endothelial phenotype [50], which occurs physiologically in the placenta after trophoblastic migration and invasion. Considering our findings, Reelin’s dual role in the placenta might initially induce trophoblastic migration during the transition from normoxia to hypoxia, possibly by forming a chemotactic gradient from nearby secreting cells, as occurs in neurons [82–85]. Subsequently, upon restoration of normoxia in the second trimester, Reelin could inhibit trophoblastic migration and invasion by acting as a stop signal [86], promoting EVT endothelial differentiation [87], and remodeling the uterine vasculature. Thus, Reelin signaling would be relevant to support a healthy pregnancy, given that deficient trophoblastic migration/invasion and remodeling of the uterine spiral arteries are hallmarks of human PE placentas [81], as discussed previously.

Regarding ApoER2 and VLDLR, we endorse their presence in term placentas. ApoER2 was found in both trophoblasts and endothelial cells. Both receptors were found in the EVT cell lines Swan 71 and HTR-8/SVneo. An interesting observation in the analyses of our results was the presence of intranuclear ApoER2 staining in the EVT lines. In neurons, Reelin influences various cellular processes [4, 5, 88]. On the one hand, it reduces receptor levels by inducing its ubiquitination via the E3 ubiquitin ligase IDOL for lysosomal degradation [89], as well as by proteolytic processing (shedding) by various metalloproteases [41, 90, 91]. After shedding, the γ-secretase complex generates an ApoER2-derived intracellular soluble fragment (ICD) [92] of 15–25 kDa [42, 93] that is translocated to the nucleus for gene expression regulation, including that of the *RELN* itself [42, 43] and for translatome regulation [94, 95]. Besides the nuclear presence of ApoER2 indicative of proteolysis, and in contrast to the canonical role of Reelin in reducing ApoER2 levels, EVTs treated with Reelin for 24 h showed a significant increase in total ApoER2 levels. This unexpected finding illustrates the interesting differences of this system compared to what is known in neurons. This effect was not explained by increased transcription, as measured by mRNA levels at 6 and 24 h of stimulation, suggesting the possibility of regulation in the translation of the ApoER2 transcript, as recently demonstrated in other systems [94–96]. In contrast, VLDLR was not increased by Reelin treatment, and its half-life was shorter than that of ApoER2, reflecting differences in protein stability and regulation. Moreover, VLDLR but not ApoER2 mRNA levels were significantly increased by CoCl_2_. In line with this result, an unpublished study from Hong et al. (2011) conducted a microarray assay of Swan 71 cells treated with CoCl_2_ showed that VLDLR were one of the most upregulated genes in the data set (https://www.ncbi.nlm.nih.gov/geo/query/acc.cgi?acc=GSE31679).

### Reelin as a potential biomarker for PE

The abnormal extension of the physiological period of hypoxia during gestation, extending to the remaining trimesters, or the occurrence of continuous cycles of hypoxia-reoxygenation (H/R) [97] leads to pregnancy pathologies such as fetal growth restriction (FGR) and PE. The latter affects between 2% and 8% of global pregnancies [98], and it is a condition characterized by poor placentation [51], which results in weakened migration and invasion of trophoblasts, reduced arterial remodeling, and oxidative stress, among other issues. Currently, PE is diagnosed after 20 weeks of gestation, as its main manifestations are hypertension (systolic blood pressure ≥ 140 mmHg and/or diastolic blood pressure ≥ 90 mmHg) and proteinuria (≥ 300 mg/24 h) [99]. When these findings are accompanied by more severe clinical or laboratory abnormalities—such as hypertensive crisis (systolic ≥ 160 mmHg or diastolic ≥ 110 mmHg), thrombocytopenia (platelet count < 100,000/µL), elevated liver transaminases, serum creatinine > 1.1 mg/dL, pulmonary edema, or persistent neurological or visual disturbances—the condition is classified as preeclampsia with severe features (PE-SF) [100]. With this in mind, we also aimed to characterize Reelin and its receptors in this pathologic condition.

ApoER2 and VLDLR were expressed in term placentas from both normal and PE-SF pregnancies. We measured full-length ApoER2 and its fragments in several term control and PE placentas and found no difference in ApoER2 levels between the two conditions. Nonetheless, since PE is presumed to develop initially during the first trimester and the placentas analyzed were obtained at birth, few conclusions can be drawn regarding the expression of the receptors and their role in the development of PE during this early period. However, Reelin was detected in term human placentas in our study and previously [21]. The glycoprotein likely originates from nearby organs, such as the ovary [101], where it promotes follicular development [102] and aromatase activity [101, 103], the intestines [104] and the liver [105, 106], all known organs for Reelin’s endocrine behavior. However, Reelin could still be produced and secreted locally, as its transcripts have been found in second-trimester and term- placentas [107]. Considering that all the placentas analyzed were obtained from third-trimester pregnancies, the variation of circulating Reelin expression across trimesters remains uncertain. However, decreased Reelin levels were observed in a second cohort of women evaluating first-trimester plasma samples from those who later developed PE-SF, a remarkable discovery given that this pathology is characterized by impaired trophoblastic migration during this period, a role that Reelin’s newly proposed function in promoting trophoblastic migration in hypoxic conditions correlates with. We need to acknowledge that the small sample size constitutes a limitation of our study, and that future studies with larger, prospectively powered cohorts will be required to test for confounding by clinical/biochemical parameters and to implement multivariable models (e.g., linear regression) and appropriate analyses of sensitivity. Then, new assays will be essential to determine whether Reelin has real predictive usefulness in early pregnancy.

Besides migration and differentiation to eEVTs, Reelin could have other roles in pregnancy related to cellular senescence. While physiological senescence is required for normal placental development as triggered through syncytiotrophoblast formation by cytotrophoblast fusion [108], oxidative stress through reactive oxygen species (ROS) production is known to induce premature placental senescence and accelerate cytotrophoblast aging, which in turn has been linked to early labor and PE through elevated senescence biomarkers [109–113]. The chronic hypoxic state that characterizes PE induces oxidative stress [114–118], and HIF-1α is a master regulator of placental senescence [119]. In this regard, our results show that Reelin treatment, in combination with CoCl_2_-hypoxia induction, downregulates this transcription factor, reinforcing the potential role of this signaling pathway in healthy placenta development and function. Likely, increased senescence has been linked with age-related neurodegenerative diseases such as Alzheimer’s and Parkinson’s [120, 121], while Reelin treatment alleviates it [122, 123]. Therefore, alongside Reelin’s recent neuroprotective denomination, an additional physiological role could be considered during normal and pathological human placentation.

Overall, the early, first-trimester downregulation of circulating Reelin in patients who would develop PE could underlie deficiencies in EVTs’ migration/invasion and in their differentiation into eEVTs, allowing inadequate remodeling of spiral arteries and, eventually, an increase in placental senescence. Thus, decreased Reelin levels could hold promise as a predictive biomarker, enabling earlier identification of women at risk of developing PE. Finally, and still relevant, is that Reelin deficit has been linked to neurodegenerative and neuropsychiatric disorders such as autism spectrum disorder (ASD) [124] and schizophrenia [125], thus recently acquiring a neuroprotective function [123, 126, 127]. In this regard, an interesting association indicates an increase in the prevalence of PE in women suffering schizophrenia [128], as well as an increased risk of developing schizophrenia in male patients whose mother suffered from PE [129].

## Conclusions

We have described that placental ApoER2 and VLDLR are expressed in extravillous trophoblasts (EVT) that respond to Reelin, increasing their migration in low O2 tension (hypoxia) and stimulating EVT differentiation to an endothelial EVT in a PI3K-dependent manner. These results suggest a role for the Reelin signaling pathway during early placentation. Both receptors and Reelin are also present in term placentas. Besides, decreased Reelin levels in first-trimester blood of pregnant women who develop PE suggest that Reelin may be a predictive biomarker for PE, linking impaired trophoblast function with defective placentation.

## Supplementary Information

Below is the link to the electronic supplementary material.

Supplementary Material 1

Supplementary Material 2

## Data Availability

The datasets during and/or analyzed during the current study are available from the corresponding author on reasonable request.
