# Supplementary material for: Reelin regulates the migration and differentiation of extravillous trophoblastic cells
Source: Biol Res. 2026 Mar 26;59:29. doi: 10.1186/s40659-026-00690-1 (PMC13141450; doi:10.1186/s40659-026-00690-1)
Supplement: Supplementary file 1 — Supplementary Material 1 [file 40659_2026_690_MOESM1_ESM.pdf]

SUPPLEMENTARY TABLES

Table S1. Antibodies used in this study				
Immunofluorescence / Immunohistochemistry				
**Antibody**	**Host**	**Catalog Number**	**Vendor**	**Dilution**
ApoER2	Rabbit	A3481	Sigma-Aldrich	1:2500<br>1:100
CD31	Mouse	3258S	Cell Signaling	1:50
CK7	Mouse	MAB3226	Sigma-Aldrich	1:30
Reelin (E4)	Mouse	AB 1157891	DSHB, Iowa	1:150
Alexa Fluor 488 anti-mouse IgG	Donkey	A21202	Thermo Fisher	1:1000
Alexa Fluor 568 anti-mouse IgG	Goat	A11004	Invitrogen	1:1000
Anti-mouse HRP	Goat	31430	Invitrogen	1:100
Western Blot				
ApoER2	Rabbit	A3481	Sigma-Aldrich	1:2500
Phospho-Akt (Ser473)	Rabbit	9271S	Cell Signalling	1:1000
Phospho-Akt (Ser473)	Rabbit	OMA103061	Invitrogen	1:1000
Akt	Rabbit	9272S	Cell Signalling	1:1000
Phospho-p44/42 MAPK (Erk1/2) (Thr202/Tyr204)	Rabbit	9101S	Cell Signalling	1:1000
ERK (pan ERK)	Mouse	610124	BD Biosciences	1:1000
VLDLR (6A6)	Mouse	MA5-24790	Invitrogen	1:500
HIF-1α	Rabbit	NB100-479	Novus Biologicals	1:1000
HRP-Anti- β-Actin HRP-conjugated	Mouse	ab49900	Abcam	1:20000
HRP-conjugated anti-rabbit IgG	Goat	111-035-144	Jackson ImmunoResearch	1:5000
HRP-conjugated anti-mouse IgG	Goat	AP124P	Sigma-Aldrich	1:5000<br>1:3000

Table S2. Primers used in this study	
ApoER2 primers	Fw 5’-GTGGCACTAGATGTGGAAGT-3’<br>Rv 5’-GTGCAACTGCTCGTCAATG-3’
VLDLR primers	Fw 5’-CCAATTCCAGTGCACAAATG-3’<br>Rv 5’- TGAACCATCTTCGCAGTCAG-3’
GAPDH primers	Fw 5’-TCATCAGCAATGCCTCCTG-3’<br>Rv 5’-GGCCATCCACAGTCTTCTG-3’
Integrin αV	Assay ID: Hs00233808_m1 (Thermo Fisher)
VE-Cadherin	Assay ID: Hs00975233_m1 (Thermo Fisher)
β-Actin	Assay ID: Hs01060665_g1 (Thermo Fisher)

Table S3. Maternal and newborn clinical characteristics.

		Third trimester samples		First trimester samples	
		**C**	**PE**	**C**	**PE**
**Maternal variable**		n = 9	n =6	n=5	n=5
	Age (year)	29,1 ± 5,3	30,5 ± 3,3	31,4 ± 1,6	34,4 ± 1,7
	Height (cm)	159 ± 3,4	159 ± 8,9	162 ± 2	163 ± 3
	Gestational age (weeks)	38,8 ± 1,3	37,8 ± 1,3	38,6 ± 0,2	36 ± 0,7*
	Weight (Kg):				
	T1	58,1± 6,3	58,3 ± 12,2	58,4 ± 2	62,2 ± 3
	T3	71,2 ± 5,9	71,8 ± 21,2	60 ± 1,8	76.4 ± 5*
	BMI (Kg/m2):				
	T1	22,8 ± 2,6	23,1 ± 4,7	22,3 ± 1	24 ± 2
	T3	28,1 ± 2,4	29,7 ± 7,9	26,4 ± 2	29,2 ± 3*
	Systolic pressure T3	117,3 ± 7,8	146 ± 9,7*	121 ± 3	160 ± 2*
	Diastolic pressure T3	72,9 ± 5,8	89,4 ± 6,4*	75 ± 3	101 ± 3
	Proteinuria (mg/24 h)	86,2 ± 31	440,1 ± 732	99 ± 12	520 ± 53*
**Newborn variable**					
	Sex (Female/Male)	5/4	4/2	2/3	2/3
	Weight (gr)	3334 ± 401	3214 ± 607,8	3262 ± 98	2249 ± 238*
	Height (cm)	48,83 ± 6,1	50,1 ± 3,2	49,8 ± 0,4	45,2 ±0,9

Data from the two cohorts used for this study: first and third trimester samples. **C:** Control; **PE**: preeclampsia. **BMI**: body mass index. Weight, BMI, and blood pressure were determined in the first (T1, 0-14 weeks of gestation) and third (T3, 28-40 weeks of gestation) trimesters of pregnancy. Data correspond to mean ± S.D. (Student's t-analysis) * p<0.05, vs values corresponding to Control vs PE.
