# Supplementary material for: Reelin regulates the migration and differentiation of extravillous trophoblastic cells
Source: Biol Res. 2026 Mar 26;59:29. doi: 10.1186/s40659-026-00690-1 (PMC13141450; doi:10.1186/s40659-026-00690-1)
Supplement: Supplementary file 2 — Supplementary Material 2 [file 40659_2026_690_MOESM2_ESM.pdf]

SUPPLEMENTARY FIGURES

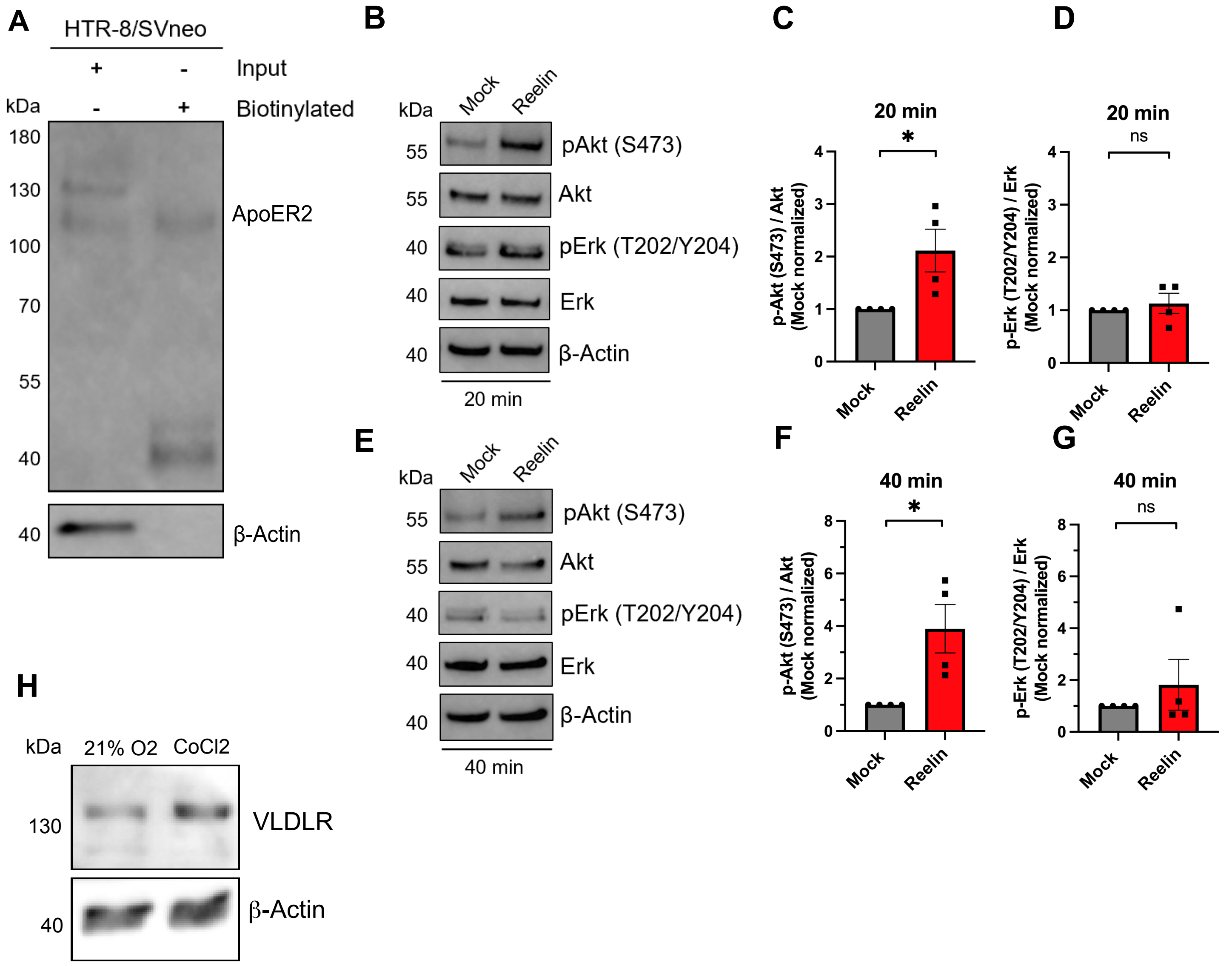

Figure S1. HTR-8/SVneo cells express ApoER2 and VLDLR and activate Reelin's canonical signaling pathway. A. Total or cell surface (biotinylated) ApoER2 levels in HTR-8/ SVneo cells treated with serum-depleted RPMI medium overnight, followed by cell surface biotinylation. 10% of the input (total receptor) and the biotinylated fraction were loaded. **B**. Representative western blot of total Akt, Erk, p-Akt (S473) and p-Erk (T202/Y204) levels are shown in HTR-8/SVneo cells treated with mock medium or 20 nM Reelin cultured in 21% O2 for 20 minutes. 40 μg of protein sample was loaded, and β-Actin was used as a loading control. **C**. Quantification of p-Akt/Akt and **D**. p-Erk/Erk levels normalized to mock values in HTR-8/ SVneo cells. Each graph corresponds to the mean ± SD of four independent experiments, and a two-way unpaired t-test was performed for all experiments (ns: $p > 0.05$ and * $p \leq 0.05$). **E**. Representative western blot of total Akt, Erk, p-Akt (S473) and p-Erk (T202/Y204) levels are shown in HTR-8/SVneo cells treated with mock medium or 20 nM Reelin cultured in 21% O2 for 40 minutes, 40 μg of protein sample was loaded and β-Actin was used as a loading control. **F.** Quantification of p-Akt/Akt and **G.** p-Erk/Erk levels normalized to mock values in HTR-8/SVneo cells. Each graph corresponds to the mean ± SD of four independent experiments, and a two-way unpaired t-test was performed for all experiments (ns: $p > 0.05$ and * $p \leq 0.05$). **H**. VLDLR immunoblot detection in extracts of Swan 71 cells cultured at 21% O2, without or with 200 μM of CoCl2 during a period of 26 h with serum-depleted medium, 80 μg of protein sample was loaded, and β-Actin was used as a loading control.

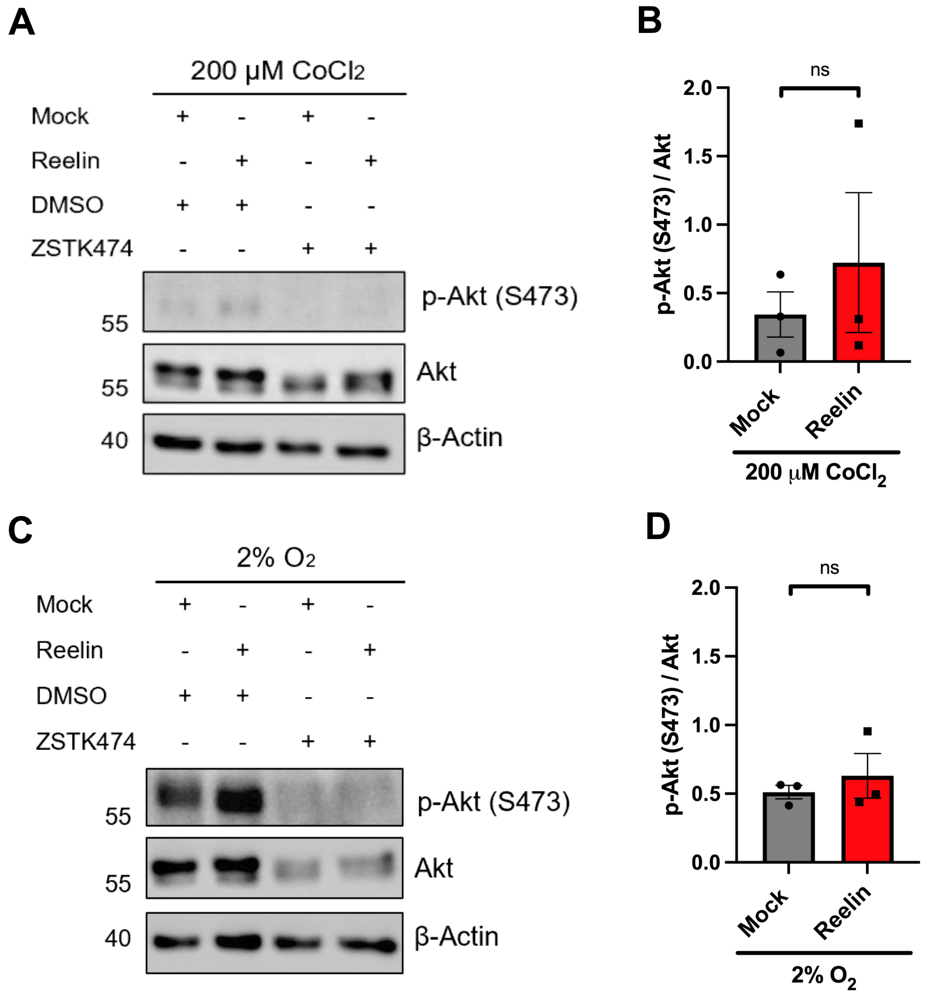

Figure S2. Effect of hypoxic conditions in Reelin-induced activation of Akt. A. Representative western blot showing total Akt and p-Akt (S473) levels detected in Swan 71 cells exposed to 24 h of 200 µM CoCl2 with 0.1 µM ZSTK474 or DMSO and mock or 20 nM Reelin, 80 µg of protein sample was loaded, and β-Actin was used as loading control. **B**. Quantification of p-Akt (S473)/Akt levels is shown in DMSO-treated Swan 71 cells. The graph represents mean ± SD of three independent experiments, and an unpaired one-tailed Welch's t-test was performed (ns: $p > 0.05$). **C.** Representative western blot showing total Akt and p-Akt (S473) levels detected in Swan 71 cells exposed to 24 h of 2% O2 with 0.1 µM ZSTK474 or DMSO and mock or 20 nM Reelin, 80 ug of protein sample was loaded and β-Actin was used as loading control. **D**. Quantification of p-Akt (S473)/Akt levels is shown in DMSO-treated Swan 71 cells. The graph represents mean ± SD of three independent experiments, and an unpaired one-tailed Welch's t-test was performed (ns: $p > 0.05$).
